# Higher frequency of prokaryotic low complexity regions in core and orthologous genes

**DOI:** 10.3389/fbinf.2025.1673480

**Published:** 2025-11-27

**Authors:** Vineet Saravanan, Alexander Kravetz, Fabia Ursula Battistuzzi

**Affiliations:** 1 Cranbrook Schools, Bloomfield Hills, MI, United States; 2 College of Literature, Science, and the Arts, University of Michigan, Ann Arbor, MI, United States; 3 Institute for Data Science, Oakland University, Rochester, MI, United States; 4 Department of Biological Sciences, Oakland University, Rochester, MI, United States

**Keywords:** low complexity region (LCR), comparative genomics, prokaryotes, pangenome, orthologs and paralogs

## Abstract

Prokaryotic genome evolution is shaped by mutation, gene duplication, and horizontal gene transfer, yet the interaction of these mechanisms, particularly in relation to low complexity regions (LCRs), remains poorly understood. LCRs are known to be mutation-prone and have been proposed to promote genetic innovation. However, the interaction between LCR-mediated and paralogy-mediated genetic innovation is still unclear. To clarify the interplay between these two evolutionary forces, we analyzed the distribution of LCRs in protein-coding genes from three closely related enterobacteria (*Escherichia coli*, *Salmonella enterica*, and *Klebsiella pneumoniae*) at both species and population levels. Using pangenomic and orthology-based approaches, we categorized genes by duplication history and conservation status and assessed LCR frequencies across these groups. We found that LCRs were consistently enriched in core and orthologous genes rather than in accessory or paralogous ones. This pattern was stable across evolutionary timescales and particularly pronounced in genes involved in cell cycle control and defense. These results suggest that, contrary to prior assumptions, LCRs may serve conserved functional roles rather than acting primarily as agents of evolutionary plasticity even at population-level timescales.

## Introduction

Three major evolutionary forces that shape prokaryote genomes include the gradual accumulation of mutations, the acquisition of genes through horizontal gene transfer, and, to a lesser extent, gene duplications (Ochman et al., 2000; Ochman, 2003; Sela et al., 2016; Abby and Vincent, 2007; Lynch et al., 2023). Through these mechanisms over billions of years, prokaryotes have evolved into the large variety of population and species known today (Poole et al., 2003; Distin, 2023). While the relative frequency of these three evolutionary mechanisms has been studied before, the intersection of these three mechanisms is poorly understood (Ochman et al., 2000; Persi et al., 2023). For example, it is well known that mutational hotspots exist and that they are often located in low complexity regions (LCRs) (Haerty and Brian Golding, 2011; Teekas et al., 2024). However, the evolutionary fate of these LCRs in duplicated or shared (i.e., horizontally transferred or evolutionarily inherited) genes is unknown.

Despite being known primarily from eukaryotes, LCRs are also present in prokaryotes, albeit in lower percentages, and are part of protein coding genes (Ntountoumi et al., 2019; Persi et al., 2023). The often repetitive structure of these regions is conducive to high mutation rates, which have led to the hypothesis of these regions being evolutionary “tuning knobs” (King et al., 1997; Kashi et al., 1997; King, 2012). The potential role of LCRs in the evolution of genomic diversity leads to two testable hypotheses: (i) the relative rate of LCRs in paralogs and ortholog should differ because of the different selective pressures acting on duplicated genes; and (ii) genes that are widely shared and conserved within and across species (core genes) should have a lower frequency of LCRs compared to those that are more sparsely distributed and more variable (accessory genes). These two hypotheses stem from a fairly straightforward scenario that correlates the high mutational rates of LCRs with gene duplication and gene sharing trends, which are driven by sequence similarity.

The availability of both population and species level data for prokaryotes offers an opportunity to test these hypotheses while also accounting for evolutionary time. The population/species boundary is expected to reflect a potential shift in evolutionary processes, from high recombination/low divergence within species to lower recombination/higher divergence among species (Touchon et al., 2009). Pangenomics has been shown to be a powerful approach to explore these trends by identifying core, conserved genes vs. accessory, more variable genes in organisms that belong to the same species or higher taxonomic units. Then, pairing this approach with orthology-detection methods that categorize genes as single or multi-copy provides the data necessary to obtain a full picture of how mutational rates in LCRs and gene duplications synergistically contribute to genomic diversity.

In this study, we apply this approach to a well-known system of three Enterobacteria species (*Escherichia coli*, *Salmonella enterica*, and *Klebsiella pneumoniae*) and their respective strains. Enterobacteria are well known species with functionally diverse strains that include both free-living and pathogenic organisms with fully sequenced genomes (Sandle, 2014). Their importance in human health, environmental microbiology, and industrial applications is well-established, thus making this work on their evolutionary plasticity significant in multiple areas of biology and beyond. By analyzing LCRs in duplicated and accessory genes, we found that the expected correlation between LCRs and high genomic diversity is not present at either the population or multi-species level. Instead, we found that LCRs are more common in orthologs and conserved (core) genes, suggesting that these regions may play a stronger functional role compared to their proposed role as “tuning knobs.”

## Methods

We obtained the complete proteomes for *Escherichia coli str. K-12 substr. MG1655*, *Salmonella enterica subsp. Enterica* serovar Typhimurium str*. LT2*, and *Klebsiella pneumoniae subsp. pneumoniae HS11286* from NCBI. For the species-level analyses, we used the reference genome for each species (ASM584v2, ASM694v2, and ASM24018v2), and for the population level analyses we randomly selected 30 genomes in each species to create three datasets of 10 strains each (Supplementary Table S1). For each species and population-level dataset, we calculated LCRs using the program SEG with default parameters (window size 12, K1 1.9, K2 2.2) (Wootton, 1994; Wootton and Scott, 1993). These parameters are commonly used to mask LCR regions during comparative analyses and, therefore, identify the most commonly identified LCRs. Additionally, these parameters identify both homo and hetero-polymers with average LCR purity (Teekas et al., 2024; Battistuzzi et al., 2016). We used ProteinOrtho with default parameters to categorize each gene as ortholog/paralog and core/accessory (Lechner et al., 2011). Genes belonging to the same ortho group and present in all 3 species (or 99% of the population genomes) were classified as core. Each gene was therefore classified in three ways: with or without LCRs, ortholog or paralog, core or accessory (Supplementary Table S2). We then calculated the frequency of LCRs as the ratio of the number of proteins with at least 1 LCR over the total number of proteins for a given category. We used chi-square tests to determine the significance of the differences observed in LCR frequencies in LCR and non-LCR containing proteins. Finally, we obtained the COG functional categories of each gene using EggNog-mapper (Cantalapiedra et al., 2021; Galperin et al., 2024) and calculated the statistical significance of over/under representation of genes with and without LCRs using a chi-square test. For each category (core, accessory, ortholog, paralog) we compared the distribution of genes with and without LCRs among different COG categories and determined its statistical significance with a chi-square test.

## Results

Genes can be categorized in two ways: (i) orthologs or paralogs, based on speciation or gene duplication histories; and (ii) core or accessory, based on their distribution among lineages. Ultimately, both of these categorizations are dependent on sequence similarity which determines how genes are related to each other within and among genomes. The presence of low complexity regions can affect their sequence similarity, as these regions are likely to have faster evolutionary rates, which results in lower sequence similarity. Thus, to determine possible correlations among LCRs, homology, and gene conservation, we performed a series of analyses at the population and species levels of *E. coli*, *S. enterica*, and *K. pneumoniae*.

First, we analyzed the three reference species for each of the species. Of the three genomes, *E. coli* and *S. enterica* are the most similar to each other as expected based on their evolutionary history (total number of proteins: 4,298 – 4,548), while *K. pneumoniae* has a larger genome (number of proteins: 5,779). Thus, relative to the total pangenome size of these three species, they contribute from 29% to 40% (Table 1).

**TABLE 1:** Species-level pangenome sizes.

Species	# Of proteins	Pangenome %
*Escherichia coli*	4,298	29.4
*Salmonella enterica*	4,548	31.1
*Klebsiella pneumoniae*	5,779	39.5
Total	14,625	100

Pangenome % represents that contribution of each species to the total pangenome (core+accessory genes).

The pangenome of these three species is composed of an approximately similar amount of core (55%) and accessory (45%) genes. However, this almost-even split changes when only protein-coding genes containing LCRs are analyzed. In this dataset, proteins containing LCRs are significantly more represented in core than in accessory genes (59% vs. 41%, chi-square test p-value <0.01) (Table 2). This result seems at odds with the high mutation rate of LCRs, which would be more likely to lead to lower genetic similarity and, therefore, higher probability of being in the accessory category.

**TABLE 2:** Percentages of pangenome core and accessory genes with or without LCRs.

Pangenomic grouping	All proteins	Proteins with LCRs	Proteins without LCRs
Pangenome	14,625	14.02% (2,051)	85.98% (12,574)
Core	54.97% (8,039)	58.8% (1,206)	54.34% (6,833)
Accessory	45.03% (6,586)	41.2% (845)	45.66% (5,741)

This result could be explained by a long evolutionary process that filtered out the most variable LCRs, thus reducing their presence in accessory genes (Persi et al., 2023; Vishnoi et al., 2010). To test this possibility, we repeated these analyses at the population level of each species. As expected, the ratio of core-to-accessory genes is higher (>70% core, <30% accessory) reflecting the higher genetic similarity within a species. However, even in these cases, the percentages of core genes with LCRs are significantly higher (74%–80%) than those of accessory genes (20%–26%) (chi-square p-values << 0.01 for each species independently) (Table 3). These results show that higher percentage of core genes with LCRs is the same at the species and population levels, suggesting that evolutionary time does not play a role in this trend.

**TABLE 3:** Population pangenome size, core, and accessory gene percentages. Data for genes with and without low complexity regions (LCRs) is shown. Ranges for three random population datasets for each species are shown in parenthesis.

Pangenomic grouping	*E. coli*	*S. enterica*	*K. pneumoniae*
Pangenome size	47,689 (47,000–48190)	43,322 (42,279–45392)	49,264 (48,632–49781)
Core	69.7% (66%–73.5%)	73.5% (72.7%–75%)	76% (72.6%–78.7%)
Accessory	30.3% (26.5%–34%)	27% (25%–27.3%)	24 (21.3%–27.4%)

	LCRs	Non-LCRs	LCRs	Non-LCRs	LCRs	Non-LCRs
Core	74.3% (69.5%–78.4%)	69.1% (65.5%–72.9%)	79.6% (78.2%–81.7%)	72.6% (71.6%–74.4%)	80.2 (77.3%–82.9%)	75.12% (71.7%–77.9%)
Accessory	25.7% (21.6–30.5)	30.9% (27.1%–34.5%)	20.4% (18.3%–21.8%)	27.4% (25.6%–28.4%)	19.8% (17.1%–22.7%)	24.8% (22.1%–28.3%)

We repeated similar analyses with genes categorized as orthologs and paralogs. These two categories of genes are known to evolve under different selective pressures, with orthologs being more conserved and paralogs, especially recent ones, experiencing relaxed purifying selection which leads to higher evolutionary rates (Koonin, 2005; Ahrens et al., 2020). Thus, it can be hypothesized that the higher evolutionary rates of paralogs could be obtained through the accumulation of mutations in LCRs. Interestingly, we found that this was not the case. Instead, we found a higher probability of genes with LCRs in the ortholog category than in paralogs (chi-square test p-values << 0.01), again supporting the hypothesis that genes with LCRs are more likely to be conserved than those without LCRs. It is also possible that the relative lengths of proteins and LCRs in orthologs and paralogs is different, leading to a different evolutionary behavior of LCRs in these two categories. To test this possibility, we performed Mann Whitney U tests on the LCR lengths of orthologs vs. paralogs and found a significant difference (p-value 0.006). However, upon closer examination, we found that this result is driven purely by 23 LCRs in a single gene (StfR) which is a putative prophage side tail fiber (NP_415890.2). Upon elimination of this outlier, the p-value rises to 0.82 showing that there is no statistically significant difference in LCR length between orthologs and paralogs. Similarly, the protein length between orthologs and paralogs is not statistically significantly different (p-value 0.06 and 0.28 without StfR).

**FIGURE 1:**
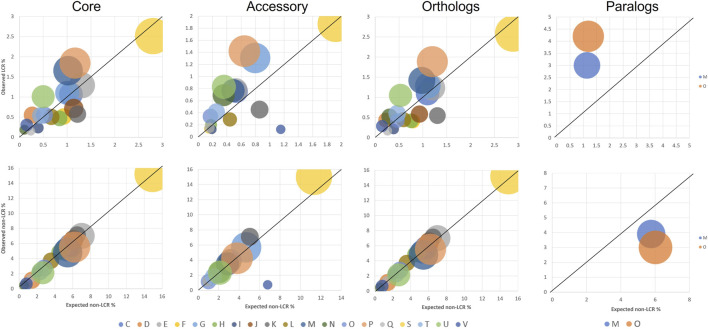
Representation of COG functional categories in genes with LCRs (top row) and without LCRs (bottom row). Circle diameters represent the number of LCR-containing genes in each category. Genes are analyzed independently based on their categorization as core or accessory, orthologs or paralogs. In each category, only COG categories with at least 10 LCR genes are shown. All distributions between LCR and non-LCR genes are significantly different (chi-square p-value <<0.01). Values on the axes are normalized to the total number of genes analyzed (core: 8,517; accessory: 7,958; orthologs: 10,759; paralogs: 333).

From a functional perspective, core/accessory and orthologous/paralogous genes have different distributions among COG categories. To identify potential correlations between COG functions and LCR distributions, we investigated the patterns in functional category for genes with and without LCRs. We performed this analysis at the species level as gene functions within species are largely conserved (Santoni and Romano-Spica, 2009). The overall distribution of genes with and without LCRs is significantly different across COG functional categories in each group of core, accessory, orthologs, and paralogs (chi-square p-values << 0.01 in each group). Some categories show clear trends of enrichment or depletion in LCR vs. non-LCR genes: enriched in LCRs are D (Cell cycle control, cell division, chromosome partitioning), N (Cell motility), O (Post-translational modification, protein turnover, chaperon functions), P (Inorganic ion transport and metabolism), T (Signal transduction mechanisms), and U (Intracellular trafficking, secretion, and vesicular transport); depleted are F (Nucleotide metabolism and transport), I (Lipid metabolism), J (Translation, ribosomal structure and biogenesis), K (Transcription), L (Replication, recombination, and repair), Q (Secondary structure), and also the unknown function (S) (Figure 1). These functional associations are similar to those previously found, in particular with LCRs being involved in transport, signal transduction, and trafficking, genes that are evolutionarily dynamic (Wright and Jane Dyson, 2015; Dyson and Wright, 2005; Puigbò et al., 2014). However, within LCR-containing genes specifically, two functional categories (D and V (Defense mechanisms)) are enriched in core and orthologs, and four are enriched only in accessory and/or paralogs (C (Energy production and conversion), E (Amino acid metabolism and transport), H (Coenzyme metabolism and transport), and O (Post-translational modification, protein turnover, chaperon functions)) (Figure 1).

## Discussion

The accumulation of mutations and the acquisition/loss/duplication of genes among genomes are primary mechanisms that lead to genomic diversity. While comparative genomics has been traditionally based on sequence similarity, the concept of a pangenome expanded this approach to include gene presence/absence as a measure of similarity among genomes. Nonetheless, pangenomes still rely on sequence similarity measures to cluster genes and, therefore, provide a way to connect these two mechanisms: from genes that are highly similar and shared across multiple lineages (core) to genes that are so different that they are not clustered together, thus forming groups of accessory genes (i.e., missing in some of the genomes). Additionally, core and accessory genes can also be either single or multi-copy, which also can affect sequence similarity by altering selective pressures on different copies (Innan and Kondrashov, 2010; Kondrashov, 2012; Qian and Zhang, 2014; Vishnoi et al., 2010). The manifestation of all these different evolutionary mechanisms is a change in sequence similarity. However, this change can be small, especially when observing population level data. Thus, it is convenient to focus on regions in a genome with high mutation rates, such as low complexity regions. In addition to being highly variable, these regions have also been proposed as key players in evolutionary innovations (“tuning knobs”), which would suggest a more widespread presence in specialized (i.e., non-conserved) genes (Kashi et al., 1997; King, 2012).

To test this prediction, we analyzed population and species level data for three closely related bacteria, *E. coli, S. enterica*, and *K. pneumoniae*, to determine the patterns of association between LCRs and sequence conservation. As a proxy for sequence conservation, we used orthology/paralogy and core/accessory categorizations. Contrary to the expectation of an association between LCR and low sequence conservation (accessory and paralogous genes), our results show that both at the species and population level, LCRs are primarily present in core and orthologous genes, which are most likely to be more conserved than accessory and paralogs (Koonin, 2005; Jordan et al., 2002). The presence of the same trend at different timescales (populations and species) suggests that this is a functionality-driven pattern rather than the result of time leading to the filtering of highly divergent sequences. Thus, this result suggests one primary conclusion: LCRs are important elements in conserved proteins. The association of LCRs with orthologs supports a recent a study that proposed LCRs and gene duplication as two sequential processes leading to genomic diversity (Persi et al., 2023). However, our results expand this model by showing a preferential association of LCRs with core and orthologous genes even at the population level, thus suggesting that the separation of the LCR and gene duplication evolutionary mechanisms happens very early in the history of a species.

Interestingly, the core and orthologous genes with high LCR frequency are primarily in the cell cycle and defense functional categories, while those in the accessory and paralogous genes are in metabolism, post-translational modifications, and energy production functions. This result could be explained in different ways. For example, it is possible that once a gene has obtained its function, the LCRs embedded in the gene lose their high variability in favor of maintaining the function of the gene. However, this interpretation disagrees with the known lower conservation of genes especially in the defense category (Makarova et al., 2011; Makarova et al., 2013). Thus, an alternative interpretation is that LCRs in these genes actually maintain their high variability rate and provide necessary diversity to genes that require constant adaptation but that are otherwise conserved (Kashi et al., 1997; King, 2012; Velasco and Hernández-Morales, 2013; King and Kashi, 2007). The two scenarios are not mutually exclusive and are likely to explain different subsets of LCRs. Irrespective of these scenarios, the similarity of our results at both population and species level suggests that the functional role of the LCRs is gained relatively quickly after their evolution and is then maintained over time. Future analyses that quantify the conservation of genes within and outside of their LCRs will allow to determine the specific functional role played by these regions.

## Data Availability

Publicly available datasets were analyzed in this study. This data can be found here: https://www.ncbi.nlm.nih.gov/, ASM584v2, ASM694v2, and ASM24018v2.
