# Supplementary material for: Higher frequency of prokaryotic low complexity regions in core and orthologous genes
Source: Front Bioinform. 2025 Nov 27;5:1673480. doi: 10.3389/fbinf.2025.1673480 (PMC12695832; doi:10.3389/fbinf.2025.1673480)
Supplement: Supplementary file 1 [file DataSheet2.pdf]

Supplementary table S2

		LCR sizes				Core Orthologs	Core Paralogs	Accessory Orthologs	Accessory Paralogs
K. pneumoniae	YP_005228646.1	14				0	1	0	0
E. Coli	NP_415146.1	13				0	1	0	0
E. Coli	NP_416699.1	15				0	1	0	0
S. enterica	NP_461191.1	15				0	1	0	0
S. enterica	NP_462713.1	15				0	1	0	0
K. pneumoniae	YP_005227354.1	9				0	1	0	0
E. Coli	NP_415149.4	13				0	1	0	0
E. Coli	NP_416704.1	13				0	1	0	0
S. enterica	NP_461196.1	9				0	1	0	0
S. enterica	NP_462718.1	9				0	1	0	0
K. pneumoniae	YP_005227359.1	12	11	14		0	1	0	0
E. Coli	NP_416564.4	11				0	1	0	0
E. Coli	NP_415501.1	12				0	1	0	0
K. pneumoniae	YP_005227868.1	14				0	1	0	0
E. Coli	NP_415003.1	13				0	1	0	0
K. pneumoniae	YP_005225497.1	35				0	1	0	0
E. Coli	NP_416700.1	12	24			0	1	0	0
S. enterica	NP_461192.1	12	18	14		0	1	0	0
S. enterica	NP_462714.1	12	18	14		0	1	0	0
K. pneumoniae	YP_005227355.1	11	11	11		0	1	0	0
S. enterica	NP_459063.1	11				0	1	0	0
K. pneumoniae	YP_005225044.1	11				0	1	0	0
E. Coli	NP_414691.1	16	17	17		0	1	0	0
S. enterica	NP_459195.1	11	15	1	13	0	1	0	0
K. pneumoniae	YP_005225197.1	15	7	16		0	1	0	0
K. pneumoniae	YP_005225165.1	18				1	0	0	0
K. pneumoniae	YP_005227674.1	15				1	0	0	0
E. Coli	NP_415567.1	9				1	0	0	0
S. enterica	NP_460122.1	9				1	0	0	0
K. pneumoniae	YP_005226240.1	9				1	0	0	0
E. Coli	NP_417063.1	7				1	0	0	0
E. Coli	NP_418635.1	14				1	0	0	0
S. enterica	NP_463265.1	14				1	0	0	0
K. pneumoniae	YP_005224753.1	14				1	0	0	0
E. Coli	NP_414560.1	13	15			1	0	0	0
S. enterica	NP_459044.1	19				1	0	0	0
K. pneumoniae	YP_005225026.1	13				1	0	0	0
E. Coli	NP_417542.1	21				1	0	0	0
S. enterica	NP_462129.1	27				1	0	0	0
K. pneumoniae	YP_005228933.1	18				1	0	0	0
E. Coli	NP_415970.4	11				1	0	0	0
S. enterica	NP_460543.1	11				1	0	0	0
E. Coli	NP_415022.1	13				1	0	0	0

S. enterica	NP_459496.1	13			1	0	0	0
K. pneumoniae	YP_005225515.1	13			1	0	0	0
K. pneumoniae	YP_005229481.1	13	9		1	0	0	0
E. Coli	NP_417153.1	15			1	0	0	0
K. pneumoniae	YP_005229310.1	14			1	0	0	0
E. Coli	NP_415615.1	17			1	0	0	0
S. enterica	NP_460169.1	6			1	0	0	0
K. pneumoniae	YP_005228694.1	15			1	0	0	0
S. enterica	NP_459813.1	8			1	0	0	0
K. pneumoniae	YP_005225980.1	14			1	0	0	0
K. pneumoniae	YP_005227252.1	11			1	0	0	0
E. Coli	NP_417080.4	19			1	0	0	0
S. enterica	NP_461587.1	19			1	0	0	0
K. pneumoniae	YP_005228280.1	19			1	0	0	0
E. Coli	NP_418642.1	15			1	0	0	0
K. pneumoniae	YP_005224759.1	14			1	0	0	0
E. Coli	NP_418610.4	8			1	0	0	0
K. pneumoniae	YP_005224729.1	12			1	0	0	0
E. Coli	NP_414998.1	13	11		1	0	0	0
S. enterica	NP_459473.1	14	1	13	1	0	0	0
K. pneumoniae	YP_005225491.1	14	1	13	1	0	0	0
S. enterica	NP_462481.1	12			1	0	0	0
E. Coli	NP_415329.4	9	1		1	0	0	0
S. enterica	NP_459804.1	15	1		1	0	0	0
K. pneumoniae	YP_005225969.1	6	12	1	1	0	0	0
K. pneumoniae	YP_005225187.1	13			1	0	0	0
E. Coli	NP_416045.1	14	12	15	1	0	0	0
S. enterica	NP_460482.1	11	9		1	0	0	0
K. pneumoniae	YP_005226851.1	14	22		1	0	0	0
E. Coli	NP_417980.1	16			1	0	0	0
S. enterica	NP_462510.1	16			1	0	0	0
K. pneumoniae	YP_005229329.1	16			1	0	0	0
K. pneumoniae	YP_005225415.1	11			1	0	0	0
K. pneumoniae	YP_005224987.1	11			1	0	0	0
E. Coli	NP_418282.1	11			1	0	0	0
S. enterica	NP_462860.1	11			1	0	0	0
K. pneumoniae	YP_005224479.1	11			1	0	0	0
S. enterica	NP_460959.1	1	11		1	0	0	0
K. pneumoniae	YP_005227805.1	1			1	0	0	0
S. enterica	NP_463138.1	16			1	0	0	0
K. pneumoniae	YP_005224616.1	14			1	0	0	0
E. Coli	NP_416151.1	12			1	0	0	0
E. Coli	NP_415477.1	12			1	0	0	0
S. enterica	NP_460044.1	12			1	0	0	0
K. pneumoniae	YP_005226165.1	15			1	0	0	0
K. pneumoniae	YP_005228014.1	16			1	0	0	0
E. Coli	NP_415297.1	14	11		1	0	0	0

S. enterica	NP_459773.1	11			1	0	0	0
K. pneumoniae	YP_005228146.1	13			1	0	0	0
E. Coli	NP_418208.1	11			1	0	0	0
S. enterica	NP_462784.1	11			1	0	0	0
K. pneumoniae	YP_005224311.1	11			1	0	0	0
E. Coli	NP_418302.1	13			1	0	0	0
S. enterica	NP_462883.1	17	14		1	0	0	0
K. pneumoniae	YP_005224323.1	12			1	0	0	0
E. Coli	NP_415623.1	11			1	0	0	0
S. enterica	NP_460177.1	18			1	0	0	0
K. pneumoniae	YP_005226281.1	16			1	0	0	0
E. Coli	NP_418408.1	11			1	0	0	0
E. Coli	NP_418430.2	19			1	0	0	0
S. enterica	NP_463041.1	2	25		1	0	0	0
K. pneumoniae	YP_005226819.1	9			1	0	0	0
S. enterica	NP_461753.1	11			1	0	0	0
K. pneumoniae	YP_005228408.1	11			1	0	0	0
E. Coli	NP_415628.1	17			1	0	0	0
S. enterica	NP_460182.1	17			1	0	0	0
K. pneumoniae	YP_005226286.1	19			1	0	0	0
S. enterica	NP_460618.1	12			1	0	0	0
K. pneumoniae	YP_005226738.1	12			1	0	0	0
S. enterica	NP_463192.1	11	14	7	1	0	0	0
K. pneumoniae	YP_005224677.1	12			1	0	0	0
E. Coli	NP_417397.1	12			1	0	0	0
S. enterica	NP_461981.1	12			1	0	0	0
K. pneumoniae	YP_005228716.1	2			1	0	0	0
K. pneumoniae	YP_005227667.1	1			1	0	0	0
E. Coli	NP_418782.1	11			1	0	0	0
K. pneumoniae	YP_005226120.1	1	9		1	0	0	0
S. enterica	NP_461275.1	12			1	0	0	0
E. Coli	NP_417925.1	8	14		1	0	0	0
S. enterica	NP_462476.1	6			1	0	0	0
E. Coli	NP_418381.1	11			1	0	0	0
E. Coli	NP_418195.2	18			1	0	0	0
S. enterica	NP_462771.1	18			1	0	0	0
K. pneumoniae	YP_005229614.1	19	8		1	0	0	0
K. pneumoniae	YP_005229588.1	11			1	0	0	0
E. Coli	NP_416905.1	11			1	0	0	0
S. enterica	NP_461360.2	11			1	0	0	0
K. pneumoniae	YP_005228122.1	11			1	0	0	0
E. Coli	NP_417759.1	2			1	0	0	0
S. enterica	NP_462324.1	2			1	0	0	0
K. pneumoniae	YP_005229148.1	22			1	0	0	0
S. enterica	NP_462151.1	13			1	0	0	0
S. enterica	NP_460649.1	11			1	0	0	0
S. enterica	NP_459699.1	15			1	0	0	0

S. enterica	NP_462821.1	1			1	0	0	0
E. Coli	NP_418561.1	12			1	0	0	0
S. enterica	NP_463189.1	12			1	0	0	0
K. pneumoniae	YP_005224673.1	12	14		1	0	0	0
S. enterica	NP_461917.1	11			1	0	0	0
K. pneumoniae	YP_005228598.1	8	11		1	0	0	0
S. enterica	NP_462120.3	11			1	0	0	0
E. Coli	NP_417418.1	11			1	0	0	0
S. enterica	NP_462007.1	11			1	0	0	0
K. pneumoniae	YP_005228745.1	11			1	0	0	0
E. Coli	NP_415398.1	1			1	0	0	0
E. Coli	NP_416468.1	12	12		1	0	0	0
E. Coli	NP_415004.1	16			1	0	0	0
S. enterica	NP_459480.1	16			1	0	0	0
K. pneumoniae	YP_005225499.1	16			1	0	0	0
E. Coli	NP_416439.1	11			1	0	0	0
K. pneumoniae	YP_005226710.1	13			1	0	0	0
K. pneumoniae	YP_005227420.1	14			1	0	0	0
E. Coli	NP_415255.1	22			1	0	0	0
K. pneumoniae	YP_005225870.1	21			1	0	0	0
K. pneumoniae	YP_005228705.1	15			1	0	0	0
E. Coli	NP_418489.1	11			1	0	0	0
K. pneumoniae	YP_005224606.1	11			1	0	0	0
K. pneumoniae	YP_005225098.1	18			1	0	0	0
E. Coli	NP_416866.1	11			1	0	0	0
S. enterica	NP_462702.1	11			1	0	0	0
K. pneumoniae	YP_005229553.1	11			1	0	0	0
E. Coli	NP_417957.1	12			1	0	0	0
S. enterica	NP_462498.1	12			1	0	0	0
K. pneumoniae	YP_005229321.1	11			1	0	0	0
E. Coli	NP_415785.1	12			1	0	0	0
S. enterica	NP_460678.1	11			1	0	0	0
K. pneumoniae	YP_005228597.1	15			1	0	0	0
E. Coli	NP_418627.4	18			1	0	0	0
E. Coli	NP_418444.1	12	13		1	0	0	0
S. enterica	NP_463054.1	12	13		1	0	0	0
K. pneumoniae	YP_005224534.1	14	13		1	0	0	0
K. pneumoniae	YP_005224963.1	8			1	0	0	0
S. enterica	NP_459489.1	9			1	0	0	0
K. pneumoniae	YP_005228950.1	11			1	0	0	0
E. Coli	NP_415270.1	9			1	0	0	0
S. enterica	NP_459735.1	9			1	0	0	0
K. pneumoniae	YP_005225884.1	9	18	16	1	0	0	0
E. Coli	NP_418448.1	11			1	0	0	0
S. enterica	NP_463085.1	11	1		1	0	0	0
E. Coli	NP_415121.1	13	13	19	1	0	0	0
S. enterica	NP_460440.1	14			1	0	0	0

K. pneumoniae	YP_005226734.1	12			1	0	0	0
E. Coli	NP_417174.1	19			1	0	0	0
S. enterica	NP_461745.1	16			1	0	0	0
E. Coli	NP_415799.1	11			1	0	0	0
S. enterica	NP_460664.1	11			1	0	0	0
K. pneumoniae	YP_005226487.1	11			1	0	0	0
E. Coli	NP_416331.1	25			1	0	0	0
S. enterica	NP_460786.1	24			1	0	0	0
K. pneumoniae	YP_005227647.1	25			1	0	0	0
K. pneumoniae	YP_005225607.1	17			1	0	0	0
S. enterica	NP_462454.1	13			1	0	0	0
E. Coli	NP_417884.1	17			1	0	0	0
S. enterica	NP_462428.1	17			1	0	0	0
K. pneumoniae	YP_005229244.1	17			1	0	0	0
E. Coli	NP_416149.1	9			1	0	0	0
S. enterica	NP_460417.1	12			1	0	0	0
E. Coli	NP_417650.1	12	11	16	1	0	0	0
S. enterica	NP_462212.1	12	11	23	1	0	0	0
K. pneumoniae	YP_005229032.1	12	13	22	1	0	0	0
E. Coli	NP_417189.2	12			1	0	0	0
S. enterica	NP_461760.1	12			1	0	0	0
E. Coli	NP_417266.1	13			1	0	0	0
S. enterica	NP_461879.1	13			1	0	0	0
K. pneumoniae	YP_005228505.1	12			1	0	0	0
E. Coli	NP_417441.1	11			1	0	0	0
E. Coli	NP_416304.1	11			1	0	0	0
S. enterica	NP_460245.1	12	12		1	0	0	0
K. pneumoniae	YP_005226358.1	11			1	0	0	0
E. Coli	NP_416051.1	17			1	0	0	0
S. enterica	NP_460476.1	15			1	0	0	0
K. pneumoniae	YP_005226844.1	14			1	0	0	0
E. Coli	NP_416672.1	11			1	0	0	0
S. enterica	NP_461149.1	13			1	0	0	0
K. pneumoniae	YP_005227963.1	15	13		1	0	0	0
S. enterica	NP_462916.1	1			1	0	0	0
K. pneumoniae	YP_005229298.1	11			1	0	0	0
E. Coli	NP_417379.1	11	12		1	0	0	0
S. enterica	NP_461969.1	11	12		1	0	0	0
K. pneumoniae	YP_005228704.1	11	12		1	0	0	0
S. enterica	NP_461324.1	12			1	0	0	0
S. enterica	NP_461601.1	11			1	0	0	0
S. enterica	NP_462517.1	11			1	0	0	0
K. pneumoniae	YP_005229337.1	9			1	0	0	0
S. enterica	NP_461506.1	11			1	0	0	0
S. enterica	NP_459460.1	1			1	0	0	0
E. Coli	NP_417244.1	11			1	0	0	0
K. pneumoniae	YP_005228494.1	12			1	0	0	0

E. Coli	NP_418167.1	11		1	0	0	0
S. enterica	NP_462747.1	14		1	0	0	0
K. pneumoniae	YP_005229585.1	19		1	0	0	0
E. Coli	NP_415776.1	15		1	0	0	0
S. enterica	NP_460686.1	11		1	0	0	0
K. pneumoniae	YP_005226459.1	16		1	0	0	0
K. pneumoniae	YP_005225610.1	12		1	0	0	0
S. enterica	NP_460799.1	11	16	1	0	0	0
S. enterica	NP_459799.1	11		1	0	0	0
K. pneumoniae	YP_005224714.1	12		1	0	0	0
K. pneumoniae	YP_005224985.1	12		1	0	0	0
E. Coli	NP_417669.1	11		1	0	0	0
S. enterica	NP_462230.1	11		1	0	0	0
K. pneumoniae	YP_005229051.1	11		1	0	0	0
E. Coli	NP_415285.1	18		1	0	0	0
S. enterica	NP_459761.1	18		1	0	0	0
K. pneumoniae	YP_005225902.1	17		1	0	0	0
S. enterica	NP_461091.1	12		1	0	0	0
K. pneumoniae	YP_005227911.1	15	11	1	0	0	0
K. pneumoniae	YP_005226523.1	11		1	0	0	0
E. Coli	NP_418464.1	12		1	0	0	0
S. enterica	NP_463099.1	12	18	1	0	0	0
K. pneumoniae	YP_005224563.1	12	15	1	0	0	0
E. Coli	NP_414633.1	11	11	1	0	0	0
S. enterica	NP_459134.1	11	11	1	0	0	0
K. pneumoniae	YP_005225112.1	11	11	1	0	0	0
E. Coli	NP_417663.1	11		1	0	0	0
S. enterica	NP_462224.1	9		1	0	0	0
K. pneumoniae	YP_005229045.1	12		1	0	0	0
K. pneumoniae	YP_005228000.1	11		1	0	0	0
E. Coli	NP_416580.1	14		1	0	0	0
S. enterica	NP_461073.1	14		1	0	0	0
K. pneumoniae	YP_005227888.1	14		1	0	0	0
E. Coli	NP_415757.1	8	16	1	0	0	0
S. enterica	NP_460708.1	8		1	0	0	0
K. pneumoniae	YP_005227495.1	8		1	0	0	0
E. Coli	NP_414552.1	12		1	0	0	0
S. enterica	NP_459015.1	12		1	0	0	0
K. pneumoniae	YP_005226401.1	11		1	0	0	0
E. Coli	NP_416894.1	13		1	0	0	0
S. enterica	NP_461350.1	13		1	0	0	0
E. Coli	NP_416520.1	13		1	0	0	0
S. enterica	NP_461015.1	13		1	0	0	0
S. enterica	NP_459592.1	4		1	0	0	0
E. Coli	NP_414696.1	12		1	0	0	0
K. pneumoniae	YP_005225449.1	16		1	0	0	0
E. Coli	NP_416107.1	15		1	0	0	0

S. enterica	NP_460456.1	1				1	0	0	0
S. enterica	NP_461667.1	11				1	0	0	0
S. enterica	NP_462009.1	12				1	0	0	0
E. Coli	NP_418466.1	11				1	0	0	0
K. pneumoniae	YP_005224565.1	11				1	0	0	0
S. enterica	NP_462477.1	11				1	0	0	0
E. Coli	NP_417857.1	16	1			1	0	0	0
K. pneumoniae	YP_005229220.1	1				1	0	0	0
E. Coli	NP_417942.6	17	16			1	0	0	0
E. Coli	NP_418045.4	13				1	0	0	0
K. pneumoniae	YP_005228151.1	15	12			1	0	0	0
S. enterica	NP_462546.1	18				1	0	0	0
K. pneumoniae	YP_005229368.1	16				1	0	0	0
E. Coli	NP_418772.4	14				1	0	0	0
E. Coli	NP_414657.1	31	3	32		1	0	0	0
S. enterica	NP_459158.2	29	23	31	13	1	0	0	0
K. pneumoniae	YP_005225139.1	31	23	32	11	1	0	0	0
E. Coli	NP_417170.1	7				1	0	0	0
K. pneumoniae	YP_005228389.1	6				1	0	0	0
E. Coli	NP_416109.2	14	13			1	0	0	0
S. enterica	NP_460450.3	1	1	11		1	0	0	0
K. pneumoniae	YP_005229367.1	1				1	0	0	0
E. Coli	NP_418096.4	1				1	0	0	0
S. enterica	NP_462630.3	1				1	0	0	0
S. enterica	NP_462093.1	21				1	0	0	0
K. pneumoniae	YP_005228868.1	13				1	0	0	0
E. Coli	NP_415724.2	12				1	0	0	0
S. enterica	NP_460737.3	22				1	0	0	0
K. pneumoniae	YP_005227538.1	24				1	0	0	0
S. enterica	NP_462519.1	18				1	0	0	0
K. pneumoniae	YP_005229339.1	12				1	0	0	0
E. Coli	NP_416825.1	13				1	0	0	0
S. enterica	NP_461314.1	15	11	11		1	0	0	0
K. pneumoniae	YP_005228081.1	33				1	0	0	0
E. Coli	NP_418578.1	19				1	0	0	0
S. enterica	NP_463207.1	25				1	0	0	0
K. pneumoniae	YP_005224700.1	19				1	0	0	0
E. Coli	NP_417955.1	14				1	0	0	0
S. enterica	NP_462495.1	12				1	0	0	0
K. pneumoniae	YP_005229318.1	1				1	0	0	0
S. enterica	NP_462152.1	9	13			1	0	0	0
K. pneumoniae	YP_005224554.1	13				1	0	0	0
E. Coli	NP_416279.1	11				1	0	0	0
K. pneumoniae	YP_005225078.1	15				1	0	0	0
S. enterica	NP_461436.1	13				1	0	0	0
K. pneumoniae	YP_005228199.1	13				1	0	0	0
K. pneumoniae	YP_005226783.1	8	11	8		1	0	0	0

E. Coli	NP_415761.1	12		1	0	0	0
S. enterica	NP_462375.1	11		1	0	0	0
S. enterica	NP_462811.1	17		1	0	0	0
E. Coli	NP_418348.1	13		1	0	0	0
S. enterica	NP_462940.1	13		1	0	0	0
K. pneumoniae	YP_005224372.1	15		1	0	0	0
E. Coli	NP_418161.1	15		1	0	0	0
S. enterica	NP_462742.1	13		1	0	0	0
K. pneumoniae	YP_005229581.1	12		1	0	0	0
K. pneumoniae	YP_005228638.1	11		1	0	0	0
E. Coli	NP_415768.1	3	21	1	0	0	0
S. enterica	NP_460696.1	59		1	0	0	0
K. pneumoniae	YP_005227478.1	34	23	1	0	0	0
E. Coli	NP_416638.4	1	11	1	0	0	0
S. enterica	NP_461113.1	1	2	1	0	0	0
E. Coli	NP_415154.1	12		1	0	0	0
S. enterica	NP_459619.1	12		1	0	0	0
K. pneumoniae	YP_005225793.1	12	11	1	0	0	0
S. enterica	NP_462251.1	15		1	0	0	0
K. pneumoniae	YP_005229067.1	17		1	0	0	0
E. Coli	NP_416257.1	21		1	0	0	0
S. enterica	NP_460274.1	13		1	0	0	0
K. pneumoniae	YP_005226424.1	11		1	0	0	0
K. pneumoniae	YP_005226248.1	11		1	0	0	0
S. enterica	NP_459714.1	14	15	1	0	0	0
S. enterica	NP_463209.1	12		1	0	0	0
E. Coli	NP_416567.4	12		1	0	0	0
K. pneumoniae	YP_005227875.1	12		1	0	0	0
K. pneumoniae	YP_005224305.1	9		1	0	0	0
E. Coli	NP_415323.1	12		1	0	0	0
S. enterica	NP_459801.1	14		1	0	0	0
K. pneumoniae	YP_005225963.1	14		1	0	0	0
S. enterica	NP_462288.1	9		1	0	0	0
E. Coli	NP_416648.1	13		1	0	0	0
S. enterica	NP_461128.1	13		1	0	0	0
K. pneumoniae	YP_005227947.1	13		1	0	0	0
K. pneumoniae	YP_005228417.1	8		1	0	0	0
S. enterica	NP_459241.1	11		1	0	0	0
E. Coli	NP_417667.1	14		1	0	0	0
K. pneumoniae	YP_005229362.1	12		1	0	0	0
S. enterica	NP_462148.1	16		1	0	0	0
E. Coli	NP_415788.1	18	19	1	0	0	0
S. enterica	NP_460675.1	15	19	1	0	0	0
K. pneumoniae	YP_005226472.1	15	24	1	0	0	0
E. Coli	NP_418567.1	15		1	0	0	0
S. enterica	NP_463194.1	15		1	0	0	0
K. pneumoniae	YP_005224680.1	15		1	0	0	0

E. Coli	NP_418300.1	9			1	0	0	0
S. enterica	NP_462879.1	1			1	0	0	0
K. pneumoniae	YP_005224321.1	25			1	0	0	0
E. Coli	NP_417616.1	18			1	0	0	0
K. pneumoniae	YP_005228992.1	12	15		1	0	0	0
K. pneumoniae	YP_005229384.1	12			1	0	0	0
E. Coli	NP_418106.1	15			1	0	0	0
S. enterica	NP_462641.1	15			1	0	0	0
K. pneumoniae	YP_005229463.1	15			1	0	0	0
E. Coli	NP_418109.1	11			1	0	0	0
S. enterica	NP_462644.1	11			1	0	0	0
E. Coli	NP_414737.1	11			1	0	0	0
K. pneumoniae	YP_005229434.1	11			1	0	0	0
E. Coli	NP_416831.1	13	18		1	0	0	0
S. enterica	NP_461325.1	18			1	0	0	0
K. pneumoniae	YP_005228087.1	13			1	0	0	0
K. pneumoniae	YP_005229229.1	9			1	0	0	0
K. pneumoniae	YP_005227977.1	12			1	0	0	0
E. Coli	NP_415378.1	11			1	0	0	0
K. pneumoniae	YP_005226065.1	11			1	0	0	0
E. Coli	NP_415397.1	11			1	0	0	0
S. enterica	NP_459916.2	11			1	0	0	0
S. enterica	NP_461839.1	12			1	0	0	0
E. Coli	NP_418048.1	11	11		1	0	0	0
S. enterica	NP_462583.1	11			1	0	0	0
K. pneumoniae	YP_005229398.1	11			1	0	0	0
E. Coli	NP_417619.1	16			1	0	0	0
S. enterica	NP_462180.1	16			1	0	0	0
K. pneumoniae	YP_005228995.1	16			1	0	0	0
E. Coli	NP_415895.1	1			1	0	0	0
S. enterica	NP_460434.1	1			1	0	0	0
K. pneumoniae	YP_005226652.1	1			1	0	0	0
K. pneumoniae	YP_005225456.1	16			1	0	0	0
S. enterica	NP_461365.1	11			1	0	0	0
E. Coli	NP_414740.1	14	11		1	0	0	0
S. enterica	NP_459250.1	14	1		1	0	0	0
K. pneumoniae	YP_005225245.1	14	1		1	0	0	0
E. Coli	NP_416032.1	15			1	0	0	0
K. pneumoniae	YP_005228938.1	1			1	0	0	0
S. enterica	NP_460090.1	16			1	0	0	0
K. pneumoniae	YP_005225232.1	2			1	0	0	0
E. Coli	NP_418799.1	11			1	0	0	0
E. Coli	NP_418600.4	12	16	16	1	0	0	0
S. enterica	NP_463229.1	16	16		1	0	0	0
K. pneumoniae	YP_005224725.1	12	16		1	0	0	0
K. pneumoniae	YP_005226089.1	11			1	0	0	0
K. pneumoniae	YP_005225467.1	17			1	0	0	0

E. Coli	NP_415223.1	12				1	0	0	0
K. pneumoniae	YP_005225847.1	12	11	11	16	1	0	0	0
E. Coli	NP_417637.1	25	16	14		1	0	0	0
S. enterica	NP_462199.1	27	29	16	14	1	0	0	0
K. pneumoniae	YP_005229014.1	32	16			1	0	0	0
E. Coli	NP_414690.4	14				1	0	0	0
S. enterica	NP_459194.1	14				1	0	0	0
K. pneumoniae	YP_005225175.1	14				1	0	0	0
E. Coli	NP_417847.1	14	14			1	0	0	0
S. enterica	NP_462388.1	14	17	24		1	0	0	0
K. pneumoniae	YP_005229211.1	11	14	11	18	1	0	0	0
S. enterica	NP_460805.1	12				1	0	0	0
S. enterica	NP_463376.1	13				1	0	0	0
S. enterica	NP_462236.1	12				1	0	0	0
K. pneumoniae	YP_005229056.1	16				1	0	0	0
S. enterica	NP_461998.1	13				1	0	0	0
K. pneumoniae	YP_005224477.1	1				1	0	0	0
E. Coli	NP_416634.1	13	8			1	0	0	0
S. enterica	NP_461109.1	14	13			1	0	0	0
K. pneumoniae	YP_005227930.1	12	16	16	18	1	0	0	0
E. Coli	NP_417203.1	12				1	0	0	0
S. enterica	NP_461772.2	12				1	0	0	0
E. Coli	NP_416609.1	28				1	0	0	0
S. enterica	NP_461941.1	29				1	0	0	0
K. pneumoniae	YP_005225465.1	39				1	0	0	0
K. pneumoniae	YP_005229386.1	12				1	0	0	0
E. Coli	NP_414846.1	14				1	0	0	0
K. pneumoniae	YP_005225716.1	15				1	0	0	0
E. Coli	NP_417011.1	15	18			1	0	0	0
S. enterica	NP_461459.1	12	16	15		1	0	0	0
K. pneumoniae	YP_005228218.1	18				1	0	0	0
K. pneumoniae	YP_005228164.1	13				1	0	0	0
E. Coli	NP_416896.4	13				1	0	0	0
K. pneumoniae	YP_005229263.1	11				1	0	0	0
S. enterica	NP_461468.1	12				1	0	0	0
E. Coli	NP_416809.1	9				1	0	0	0
S. enterica	NP_461293.1	9				1	0	0	0
K. pneumoniae	YP_005228064.1	9				1	0	0	0
K. pneumoniae	YP_005229363.1	14				1	0	0	0
S. enterica	NP_460459.1	12				1	0	0	0
K. pneumoniae	YP_005226792.1	15				1	0	0	0
E. Coli	NP_418193.1	15				1	0	0	0
S. enterica	NP_462769.1	15				1	0	0	0
K. pneumoniae	YP_005229612.1	15				1	0	0	0
E. Coli	NP_415899.1	16				1	0	0	0
S. enterica	NP_460605.1	19				1	0	0	0
K. pneumoniae	YP_005226658.1	16				1	0	0	0

K. pneumoniae	YP_005224914.1	17		1	0	0	0
E. Coli	NP_418413.1	16		1	0	0	0
S. enterica	NP_463021.1	18		1	0	0	0
K. pneumoniae	YP_005224500.1	16		1	0	0	0
S. enterica	NP_463042.1	11	16	1	0	0	0
E. Coli	NP_417552.1	11		1	0	0	0
S. enterica	NP_459845.1	12	19	1	0	0	0
S. enterica	NP_462691.1	11		1	0	0	0
E. Coli	NP_416687.1	9		1	0	0	0
S. enterica	NP_461165.1	9		1	0	0	0
E. Coli	NP_415446.1	11		1	0	0	0
S. enterica	NP_459971.1	11		1	0	0	0
K. pneumoniae	YP_005226134.1	18		1	0	0	0
K. pneumoniae	YP_005227213.1	15		1	0	0	0
K. pneumoniae	YP_005226265.1	9		1	0	0	0
S. enterica	NP_460329.1	14	11	1	0	0	0
E. Coli	NP_417809.1	12	18	1	0	0	0
K. pneumoniae	YP_005229186.1	13		1	0	0	0
K. pneumoniae	YP_005229497.1	11		1	0	0	0
E. Coli	NP_418016.1	7		1	0	0	0
E. Coli	NP_415535.1	14		1	0	0	0
S. enterica	NP_460097.1	12		1	0	0	0
K. pneumoniae	YP_005226219.1	12		1	0	0	0
S. enterica	NP_460801.1	9		1	0	0	0
K. pneumoniae	YP_005225261.1	1		1	0	0	0
K. pneumoniae	YP_005225891.1	11		1	0	0	0
E. Coli	NP_415112.1	12		1	0	0	0
S. enterica	NP_459574.1	2		1	0	0	0
K. pneumoniae	YP_005225690.1	22		1	0	0	0
E. Coli	NP_417722.1	12		1	0	0	0
S. enterica	NP_462290.1	11		1	0	0	0
K. pneumoniae	YP_005229097.1	11		1	0	0	0
E. Coli	NP_415637.1	12		1	0	0	0
S. enterica	NP_460190.1	15		1	0	0	0
K. pneumoniae	YP_005226300.1	15		1	0	0	0
E. Coli	NP_414625.1	11		1	0	0	0
S. enterica	NP_459126.1	11		1	0	0	0
E. Coli	NP_418070.6	19		1	0	0	0
S. enterica	NP_462605.1	19		1	0	0	0
K. pneumoniae	YP_005229417.1	15	17	1	0	0	0
E. Coli	NP_416750.2	12		1	0	0	0
S. enterica	NP_461233.1	11		1	0	0	0
K. pneumoniae	YP_005228018.1	11		1	0	0	0
E. Coli	NP_415631.1	11		1	0	0	0
K. pneumoniae	YP_005226294.1	18		1	0	0	0
E. Coli	NP_414635.1	9	1	1	0	0	0
S. enterica	NP_459136.1	14		1	0	0	0

K. pneumoniae	YP_005225114.1	15			1	0	0	0
E. Coli	NP_415122.1	15	12		1	0	0	0
S. enterica	NP_459584.1	13	15		1	0	0	0
K. pneumoniae	YP_005225743.1	12	15	13	1	0	0	0
K. pneumoniae	YP_005227961.1	15			1	0	0	0
K. pneumoniae	YP_005226661.1	11			1	0	0	0
S. enterica	NP_463374.1	17	15		1	0	0	0
K. pneumoniae	YP_005226881.1	13	14		1	0	0	0
E. Coli	NP_416843.1	11			1	0	0	0
S. enterica	NP_461330.1	11			1	0	0	0
E. Coli	NP_417079.1	1			1	0	0	0
K. pneumoniae	YP_005228279.1	11			1	0	0	0
K. pneumoniae	YP_005227656.1	18			1	0	0	0
E. Coli	NP_418547.1	16			1	0	0	0
S. enterica	NP_463166.1	16			1	0	0	0
K. pneumoniae	YP_005224503.1	16			1	0	0	0
K. pneumoniae	YP_005224894.1	11			1	0	0	0
E. Coli	NP_417630.1	13			1	0	0	0
S. enterica	NP_462192.1	13			1	0	0	0
K. pneumoniae	YP_005229006.1	13			1	0	0	0
E. Coli	NP_414589.1	12			1	0	0	0
K. pneumoniae	YP_005225056.1	16			1	0	0	0
K. pneumoniae	YP_005228866.1	4			1	0	0	0
E. Coli	NP_417593.4	17			1	0	0	0
S. enterica	NP_462160.1	19			1	0	0	0
K. pneumoniae	YP_005228971.1	13			1	0	0	0
E. Coli	NP_417259.1	9			1	0	0	0
S. enterica	NP_461873.1	9			1	0	0	0
E. Coli	NP_417071.1	18			1	0	0	0
S. enterica	NP_461578.1	18			1	0	0	0
S. enterica	NP_462357.1	1	23		1	0	0	0
K. pneumoniae	YP_005229182.1	11	15		1	0	0	0
E. Coli	NP_415617.1	14			1	0	0	0
S. enterica	NP_460171.1	14			1	0	0	0
K. pneumoniae	YP_005226276.1	12			1	0	0	0
E. Coli	NP_416320.1	19			1	0	0	0
K. pneumoniae	YP_005227636.1	18			1	0	0	0
K. pneumoniae	YP_005225128.1	11			1	0	0	0
S. enterica	NP_461499.1	12			1	0	0	0
K. pneumoniae	YP_005228252.1	14			1	0	0	0
E. Coli	NP_416353.1	14			1	0	0	0
K. pneumoniae	YP_005227673.1	11			1	0	0	0
K. pneumoniae	YP_005227149.1	11	11		1	0	0	0
E. Coli	NP_415118.1	12	12		1	0	0	0
K. pneumoniae	YP_005225740.1	12			1	0	0	0
E. Coli	NP_418663.1	12			1	0	0	0
S. enterica	NP_463316.1	12			1	0	0	0

K. pneumoniae	YP_005224801.1	12				1	0	0	0
E. Coli	NP_418655.1	1				1	0	0	0
S. enterica	NP_463298.1	1				1	0	0	0
S. enterica	NP_462941.1	13				1	0	0	0
S. enterica	NP_460962.1	9				1	0	0	0
K. pneumoniae	YP_005227824.1	1				1	0	0	0
E. Coli	NP_415173.1	11				1	0	0	0
S. enterica	NP_459638.1	11				1	0	0	0
K. pneumoniae	YP_005225811.1	11				1	0	0	0
K. pneumoniae	YP_005225127.1	11	12			1	0	0	0
E. Coli	NP_418468.3	13				1	0	0	0
K. pneumoniae	YP_005224567.1	11				1	0	0	0
E. Coli	NP_415715.1	8				1	0	0	0
S. enterica	NP_460752.1	15				1	0	0	0
K. pneumoniae	YP_005227613.1	14				1	0	0	0
S. enterica	NP_462289.1	13				1	0	0	0
K. pneumoniae	YP_005229096.1	3				1	0	0	0
S. enterica	NP_459809.1	11				1	0	0	0
E. Coli	NP_416907.1	15	12	13		1	0	0	0
S. enterica	NP_461363.1	15	12	67	18	1	0	0	0
K. pneumoniae	YP_005228125.1	15	3	56		1	0	0	0
S. enterica	NP_461307.1	11				1	0	0	0
E. Coli	NP_414548.1	15				1	0	0	0
K. pneumoniae	YP_005225017.1	17				1	0	0	0
K. pneumoniae	YP_005225849.1	11				1	0	0	0
K. pneumoniae	YP_005229455.1	18				1	0	0	0
E. Coli	NP_417724.4	1				1	0	0	0
S. enterica	NP_462292.1	14				1	0	0	0
K. pneumoniae	YP_005229099.1	8				1	0	0	0
E. Coli	NP_416744.1	12				1	0	0	0
S. enterica	NP_461226.1	12				1	0	0	0
K. pneumoniae	YP_005228013.1	12				1	0	0	0
E. Coli	NP_417527.1	14				1	0	0	0
S. enterica	NP_462118.1	14				1	0	0	0
K. pneumoniae	YP_005228888.1	14				1	0	0	0
K. pneumoniae	YP_005226514.1	12				1	0	0	0
K. pneumoniae	YP_005229557.1	13				1	0	0	0
K. pneumoniae	YP_005224710.1	11				1	0	0	0
E. Coli	NP_414685.4	16				1	0	0	0
S. enterica	NP_459189.1	16				1	0	0	0
K. pneumoniae	YP_005225170.1	15				1	0	0	0
K. pneumoniae	YP_005229387.1	15				1	0	0	0
S. enterica	NP_462238.1	12				1	0	0	0
K. pneumoniae	YP_005229058.1	12				1	0	0	0
E. Coli	NP_417905.1	14	11			1	0	0	0
S. enterica	NP_462453.1	15				1	0	0	0
K. pneumoniae	YP_005229261.1	11				1	0	0	0

K. pneumoniae	YP_005229074.1	12					1	0	0	0
E. Coli	NP_417817.2	11					1	0	0	0
S. enterica	NP_462370.1	11					1	0	0	0
K. pneumoniae	YP_005229195.1	11					1	0	0	0
S. enterica	NP_460802.1	4					1	0	0	0
K. pneumoniae	YP_005227663.1	22					1	0	0	0
E. Coli	NP_416720.1	16					1	0	0	0
S. enterica	NP_461211.1	16					1	0	0	0
K. pneumoniae	YP_005228003.1	16	14				1	0	0	0
E. Coli	NP_418590.1	1					1	0	0	0
S. enterica	NP_463219.1	8					1	0	0	0
K. pneumoniae	YP_005224712.1	1					1	0	0	0
E. Coli	NP_416734.1	11					1	0	0	0
S. enterica	NP_461214.1	13					1	0	0	0
E. Coli	NP_418798.1	11					1	0	0	0
S. enterica	NP_463423.1	11					1	0	0	0
K. pneumoniae	YP_005224990.1	11					1	0	0	0
K. pneumoniae	YP_005228177.1	15					1	0	0	0
E. Coli	NP_418129.2	12					1	0	0	0
S. enterica	NP_462699.1	12					1	0	0	0
K. pneumoniae	YP_005229550.1	1	15				1	0	0	0
E. Coli	NP_415734.1	6					1	0	0	0
K. pneumoniae	YP_005227528.1	6					1	0	0	0
K. pneumoniae	YP_005226486.1	13					1	0	0	0
E. Coli	NP_416172.1	4					1	0	0	0
S. enterica	NP_460395.1	19	13				1	0	0	0
E. Coli	NP_417101.1	13					1	0	0	0
S. enterica	NP_461607.1	13					1	0	0	0
K. pneumoniae	YP_005228348.1	21					1	0	0	0
K. pneumoniae	YP_005225673.1	12					1	0	0	0
K. pneumoniae	YP_005225787.1	11					1	0	0	0
E. Coli	NP_417999.1	13					1	0	0	0
S. enterica	NP_462530.1	13					1	0	0	0
K. pneumoniae	YP_005229356.1	13					1	0	0	0
E. Coli	NP_414695.1	1	12	19	15	13	1	0	0	0
S. enterica	NP_459199.1	12	11	15	15		1	0	0	0
K. pneumoniae	YP_005225202.1	16	19	22	16	14	1	0	0	0
E. Coli	NP_415452.1	12					1	0	0	0
S. enterica	NP_460031.1	12					1	0	0	0
K. pneumoniae	YP_005226148.1	12					1	0	0	0
S. enterica	NP_459723.1	11					1	0	0	0
E. Coli	NP_418461.1	12					1	0	0	0
S. enterica	NP_463097.1	11					1	0	0	0
K. pneumoniae	YP_005224561.1	14					1	0	0	0
E. Coli	NP_415416.1	13					1	0	0	0
K. pneumoniae	YP_005226107.1	13					1	0	0	0
E. Coli	NP_415779.1	11					1	0	0	0

S. enterica	NP_460683.1	11				1	0	0	0
K. pneumoniae	YP_005226462.1	12				1	0	0	0
E. Coli	NP_414703.1	29	12			1	0	0	0
S. enterica	NP_459214.1	18	12			1	0	0	0
K. pneumoniae	YP_005225210.1	14				1	0	0	0
E. Coli	NP_418632.1	12				1	0	0	0
S. enterica	NP_463262.1	12				1	0	0	0
K. pneumoniae	YP_005224750.1	16				1	0	0	0
E. Coli	NP_417504.1	9				1	0	0	0
K. pneumoniae	YP_005229048.1	15				1	0	0	0
E. Coli	NP_418682.1	9				1	0	0	0
S. enterica	NP_463339.1	9				1	0	0	0
K. pneumoniae	YP_005224819.1	9				1	0	0	0
K. pneumoniae	YP_005224703.1	13				1	0	0	0
E. Coli	NP_418538.2	1				1	0	0	0
E. Coli	NP_416780.1	9				1	0	0	0
S. enterica	NP_461259.1	9				1	0	0	0
K. pneumoniae	YP_005228034.1	9				1	0	0	0
S. enterica	NP_463456.1	15				1	0	0	0
S. enterica	NP_460885.1	13				1	0	0	0
E. Coli	NP_418595.1	11	22	14	14	1	0	0	0
S. enterica	NP_463224.1	16	24	14		1	0	0	0
K. pneumoniae	YP_005224718.1	11	18	17	18	1	0	0	0
E. Coli	NP_414637.1	11	12			1	0	0	0
S. enterica	NP_459138.1	11	12			1	0	0	0
K. pneumoniae	YP_005225116.1	11	12			1	0	0	0
K. pneumoniae	YP_005227709.1	12				1	0	0	0
K. pneumoniae	YP_005226097.1	18				1	0	0	0
E. Coli	NP_417014.1	11				1	0	0	0
S. enterica	NP_461498.1	13				1	0	0	0
S. enterica	NP_459691.1	14				1	0	0	0
K. pneumoniae	YP_005225850.1	13				1	0	0	0
E. Coli	NP_417399.1	13				1	0	0	0
S. enterica	NP_461983.1	17	13			1	0	0	0
K. pneumoniae	YP_005228719.1	13				1	0	0	0
K. pneumoniae	YP_005227263.1	13				1	0	0	0
K. pneumoniae	YP_005224431.1	12	7			1	0	0	0
E. Coli	NP_416763.1	15				1	0	0	0
E. Coli	NP_417067.1	1				1	0	0	0
S. enterica	NP_461574.1	1				1	0	0	0
E. Coli	NP_417716.1	12				1	0	0	0
S. enterica	NP_459792.1	13				1	0	0	0
K. pneumoniae	YP_005225946.1	12				1	0	0	0
E. Coli	NP_417733.1	12				1	0	0	0
S. enterica	NP_462302.1	12				1	0	0	0
K. pneumoniae	YP_005229128.1	12				1	0	0	0
E. Coli	NP_417660.1	8				1	0	0	0

S. enterica	NP_462221.1	12									1	0	0	0
K. pneumoniae	YP_005229042.1	12									1	0	0	0
E. Coli	NP_414912.1	12									1	0	0	0
S. enterica	NP_461957.3	7									1	0	0	0
S. enterica	NP_459108.1	1									1	0	0	0
E. Coli	NP_417297.1	1									1	0	0	0
S. enterica	NP_461911.1	1									1	0	0	0
K. pneumoniae	YP_005228592.1	1									1	0	0	0
K. pneumoniae	YP_005226662.1	19									1	0	0	0
E. Coli	NP_415729.1	11									1	0	0	0
S. enterica	NP_460732.1	11									1	0	0	0
E. Coli	NP_415410.1	13	15	19	11	17	12	12	78		1	0	0	0
S. enterica	NP_459936.1	11	14	11	17	##	16				1	0	0	0
K. pneumoniae	YP_005226101.1	15	12	15	11	21	17	17	11	16	1	0	0	0
K. pneumoniae	YP_005229473.1	12									1	0	0	0
K. pneumoniae	YP_005224754.1	13									1	0	0	0
K. pneumoniae	YP_005227918.1	12									1	0	0	0
E. Coli	NP_418411.1	12									1	0	0	0
K. pneumoniae	YP_005224498.1	12									1	0	0	0
E. Coli	NP_417814.1	11									1	0	0	0
S. enterica	NP_462367.1	11									1	0	0	0
K. pneumoniae	YP_005229192.1	11									1	0	0	0
E. Coli	NP_416226.1	17									1	0	0	0
S. enterica	NP_460306.1	15	15								1	0	0	0
K. pneumoniae	YP_005227462.1	15	14								1	0	0	0
K. pneumoniae	YP_005228383.1	16									1	0	0	0
E. Coli	NP_415104.1	11	11								1	0	0	0
K. pneumoniae	YP_005224884.1	12	11	17							1	0	0	0
K. pneumoniae	YP_005227693.1	15									1	0	0	0
S. enterica	NP_459438.1	12									1	0	0	0
E. Coli	NP_414720.1	1									1	0	0	0
E. Coli	NP_416761.4	9									1	0	0	0
K. pneumoniae	YP_005229296.1	11									1	0	0	0
E. Coli	NP_416298.1	17									1	0	0	0
S. enterica	NP_460250.1	18									1	0	0	0
K. pneumoniae	YP_005226375.1	15									1	0	0	0
S. enterica	NP_463433.2	8									1	0	0	0
E. Coli	NP_416233.1	13									1	0	0	0
S. enterica	NP_460300.3	13									1	0	0	0
K. pneumoniae	YP_005227471.1	13									1	0	0	0
E. Coli	NP_415029.1	18	14								1	0	0	0
S. enterica	NP_459503.1	18									1	0	0	0
K. pneumoniae	YP_005225522.1	18	18								1	0	0	0
E. Coli	NP_416824.1	1	11								1	0	0	0
K. pneumoniae	YP_005228080.1	15									1	0	0	0
E. Coli	NP_416891.1	14									1	0	0	0
S. enterica	NP_461348.1	12	16								1	0	0	0

K. pneumoniae	YP_005228114.1	2			1	0	0	0
E. Coli	NP_418676.1	16			1	0	0	0
S. enterica	NP_463330.1	11	16		1	0	0	0
K. pneumoniae	YP_005224813.1	11	21		1	0	0	0
E. Coli	NP_415392.1	11			1	0	0	0
K. pneumoniae	YP_005226081.1	11			1	0	0	0
E. Coli	NP_418241.1	25			1	0	0	0
S. enterica	NP_462818.1	25			1	0	0	0
K. pneumoniae	YP_005224441.1	25			1	0	0	0
E. Coli	NP_416710.1	1			1	0	0	0
S. enterica	NP_461202.1	1			1	0	0	0
K. pneumoniae	YP_005228957.1	1			1	0	0	0
E. Coli	NP_415318.1	26			1	0	0	0
S. enterica	NP_459798.1	23			1	0	0	0
K. pneumoniae	YP_005225960.1	28	1		1	0	0	0
E. Coli	NP_414936.1	11			1	0	0	0
S. enterica	NP_459395.1	12			1	0	0	0
E. Coli	NP_418683.4	14			1	0	0	0
S. enterica	NP_463340.1	14			1	0	0	0
K. pneumoniae	YP_005224820.1	14			1	0	0	0
E. Coli	NP_416162.1	15	8		1	0	0	0
K. pneumoniae	YP_005227265.1	8			1	0	0	0
E. Coli	NP_417171.1	8			1	0	0	0
S. enterica	NP_461741.1	8			1	0	0	0
E. Coli	NP_417155.1	17			1	0	0	0
S. enterica	NP_461725.1	12			1	0	0	0
K. pneumoniae	YP_005228370.1	9			1	0	0	0
E. Coli	NP_416243.1	18			1	0	0	0
S. enterica	NP_460286.1	18			1	0	0	0
K. pneumoniae	YP_005226438.1	18			1	0	0	0
E. Coli	NP_414996.1	23			1	0	0	0
S. enterica	NP_459471.1	23			1	0	0	0
K. pneumoniae	YP_005225489.1	9			1	0	0	0
E. Coli	NP_415723.1	14			1	0	0	0
S. enterica	NP_460738.1	14			1	0	0	0
K. pneumoniae	YP_005227540.1	14			1	0	0	0
E. Coli	NP_417427.1	12			1	0	0	0
E. Coli	NP_418457.1	14			1	0	0	0
S. enterica	NP_463093.1	12			1	0	0	0
K. pneumoniae	YP_005224557.1	12			1	0	0	0
E. Coli	NP_416121.1	12			1	0	0	0
S. enterica	NP_460438.1	1			1	0	0	0
K. pneumoniae	YP_005226729.1	14			1	0	0	0
E. Coli	NP_415205.1	1	11	13	1	0	0	0
S. enterica	NP_459670.1	1	1		1	0	0	0
K. pneumoniae	YP_005225833.1	2			1	0	0	0
S. enterica	NP_461265.3	11			1	0	0	0

E. Coli	NP_414890.1	14				1	0	0	0
S. enterica	NP_460586.1	12				1	0	0	0
K. pneumoniae	YP_005227230.1	14				1	0	0	0
E. Coli	NP_415534.1	12				1	0	0	0
S. enterica	NP_447555.1	9				1	0	0	0
K. pneumoniae	YP_005226218.1	12				1	0	0	0
K. pneumoniae	YP_005229578.1	16				1	0	0	0
E. Coli	NP_418206.1	17	13			1	0	0	0
S. enterica	NP_462782.1	17	13			1	0	0	0
K. pneumoniae	YP_005224309.1	17	13			1	0	0	0
E. Coli	NP_417539.1	21				1	0	0	0
S. enterica	NP_462126.3	21				1	0	0	0
K. pneumoniae	YP_005228906.1	21				1	0	0	0
K. pneumoniae	YP_005226718.1	17				1	0	0	0
E. Coli	NP_415418.1	1				1	0	0	0
S. enterica	NP_459943.1	1				1	0	0	0
E. Coli	NP_415160.1	1				1	0	0	0
S. enterica	NP_459624.1	1				1	0	0	0
K. pneumoniae	YP_005229127.1	12	13			1	0	0	0
K. pneumoniae	YP_005226244.1	11				1	0	0	0
E. Coli	NP_416657.1	16				1	0	0	0
S. enterica	NP_461137.1	17				1	0	0	0
K. pneumoniae	YP_005228588.1	1				1	0	0	0
E. Coli	NP_415469.1	16				1	0	0	0
K. pneumoniae	YP_005226158.1	16				1	0	0	0
K. pneumoniae	YP_005225123.1	11				1	0	0	0
E. Coli	NP_415123.1	15	9	13		1	0	0	0
S. enterica	NP_459585.1	13	9	13		1	0	0	0
K. pneumoniae	YP_005225744.1	17	11	9	26	1	0	0	0
S. enterica	NP_461420.1	16				1	0	0	0
S. enterica	NP_460836.1	11				1	0	0	0
E. Coli	NP_416890.1	15	18			1	0	0	0
S. enterica	NP_461345.1	15	18	18		1	0	0	0
K. pneumoniae	YP_005228111.1	16	17	18		1	0	0	0
E. Coli	NP_417777.1	11				1	0	0	0
S. enterica	NP_462342.1	11				1	0	0	0
K. pneumoniae	YP_005229166.1	11				1	0	0	0
E. Coli	NP_417950.1	18				1	0	0	0
S. enterica	NP_462490.1	18				1	0	0	0
K. pneumoniae	YP_005229313.1	18				1	0	0	0
S. enterica	NP_462675.1	16				1	0	0	0
K. pneumoniae	YP_005229536.1	13				1	0	0	0
E. Coli	NP_416582.1	14				1	0	0	0
S. enterica	NP_461075.1	14				1	0	0	0
K. pneumoniae	YP_005227890.1	2	17			1	0	0	0
E. Coli	NP_417192.1	7				1	0	0	0
S. enterica	NP_461763.1	7				1	0	0	0

K. pneumoniae	YP_005228420.1	11	7	1	0	0	0
E. Coli	NP_415012.1	11		1	0	0	0
S. enterica	NP_459488.1	9	11	1	0	0	0
K. pneumoniae	YP_005225508.1	11		1	0	0	0
E. Coli	NP_415969.1	21		1	0	0	0
S. enterica	NP_460545.1	11		1	0	0	0
E. Coli	NP_415478.1	8	13	1	0	0	0
S. enterica	NP_460045.1	13		1	0	0	0
K. pneumoniae	YP_005226166.1	13		1	0	0	0
E. Coli	NP_416120.1	14		1	0	0	0
S. enterica	NP_460439.1	14	18	1	0	0	0
K. pneumoniae	YP_005226733.1	14		1	0	0	0
E. Coli	NP_417298.1	11		1	0	0	0
E. Coli	NP_418596.1	11		1	0	0	0
S. enterica	NP_463225.1	11		1	0	0	0
K. pneumoniae	YP_005224719.1	11		1	0	0	0
E. Coli	NP_418368.1	23		1	0	0	0
S. enterica	NP_462974.1	25		1	0	0	0
K. pneumoniae	YP_005224380.1	52		1	0	0	0
E. Coli	NP_416100.1	11		1	0	0	0
E. Coli	NP_414995.1	13		1	0	0	0
S. enterica	NP_459470.1	13		1	0	0	0
K. pneumoniae	YP_005225488.1	13		1	0	0	0
E. Coli	NP_415191.1	12		1	0	0	0
S. enterica	NP_459659.1	12		1	0	0	0
K. pneumoniae	YP_005225823.1	12		1	0	0	0
E. Coli	NP_415055.1	12		1	0	0	0
K. pneumoniae	YP_005225420.1	11		1	0	0	0
E. Coli	NP_417877.1	1		1	0	0	0
S. enterica	NP_462418.1	1		1	0	0	0
K. pneumoniae	YP_005229240.1	1		1	0	0	0
E. Coli	NP_417824.1	16		1	0	0	0
S. enterica	NP_462377.1	16		1	0	0	0
K. pneumoniae	YP_005229204.1	16		1	0	0	0
K. pneumoniae	YP_005226797.1	14		1	0	0	0
E. Coli	NP_414627.1	12	1	1	0	0	0
S. enterica	NP_459128.1	1		1	0	0	0
K. pneumoniae	YP_005225105.1	12	1	1	0	0	0
E. Coli	NP_415440.1	11		1	0	0	0
S. enterica	NP_459965.1	15		1	0	0	0
K. pneumoniae	YP_005226127.1	2	1	1	0	0	0
E. Coli	NP_417995.1	28		1	0	0	0
K. pneumoniae	YP_005229345.1	26		1	0	0	0
E. Coli	NP_417204.1	16		1	0	0	0
S. enterica	NP_461773.1	16		1	0	0	0
K. pneumoniae	YP_005228434.1	8		1	0	0	0
K. pneumoniae	YP_005227682.1	17		1	0	0	0

S. enterica	NP_461240.1	11		1	0	0	0
E. Coli	NP_414640.1	7		1	0	0	0
S. enterica	NP_459141.1	11		1	0	0	0
E. Coli	NP_414946.4	1		1	0	0	0
S. enterica	NP_459056.1	9		1	0	0	0
K. pneumoniae	YP_005225035.1	9		1	0	0	0
E. Coli	NP_417021.1	11		1	0	0	0
S. enterica	NP_461474.1	11		1	0	0	0
K. pneumoniae	YP_005228229.1	15	9	1	0	0	0
K. pneumoniae	YP_005228601.1	11		1	0	0	0
S. enterica	NP_462516.3	14		1	0	0	0
S. enterica	NP_460651.1	17		1	0	0	0
S. enterica	NP_461852.1	11		1	0	0	0
K. pneumoniae	YP_005228485.1	11		1	0	0	0
K. pneumoniae	YP_005224671.1	14		1	0	0	0
E. Coli	NP_415771.1	11		1	0	0	0
K. pneumoniae	YP_005226446.1	11		1	0	0	0
S. enterica	NP_459639.1	13		1	0	0	0
E. Coli	NP_414630.1	12		1	0	0	0
S. enterica	NP_459131.1	9		1	0	0	0
K. pneumoniae	YP_005225108.1	12		1	0	0	0
K. pneumoniae	YP_005226481.1	14	17	1	0	0	0
K. pneumoniae	YP_005229402.1	1		1	0	0	0
E. Coli	NP_416817.2	38		1	0	0	0
S. enterica	NP_461306.1	23		1	0	0	0
K. pneumoniae	YP_005228073.1	42		1	0	0	0
E. Coli	NP_417633.4	15		1	0	0	0
K. pneumoniae	YP_005229009.1	9		1	0	0	0
E. Coli	NP_416640.2	17		1	0	0	0
E. Coli	NP_416438.1	13		1	0	0	0
S. enterica	NP_460917.1	11		1	0	0	0
S. enterica	NP_462158.1	13		1	0	0	0
K. pneumoniae	YP_005227602.1	13		1	0	0	0
E. Coli	NP_417850.1	11		1	0	0	0
K. pneumoniae	YP_005229214.1	13		1	0	0	0
E. Coli	NP_416998.1	13		1	0	0	0
K. pneumoniae	YP_005228201.1	13	11	1	0	0	0
E. Coli	NP_418483.1	33		1	0	0	0
S. enterica	NP_463121.1	29		1	0	0	0
K. pneumoniae	YP_005224583.1	27		1	0	0	0
E. Coli	NP_418124.4	1		1	0	0	0
S. enterica	NP_462688.1	1		1	0	0	0
K. pneumoniae	YP_005229542.1	15		1	0	0	0
E. Coli	NP_416118.1	1		1	0	0	0
K. pneumoniae	YP_005227665.1	11	11	1	0	0	0
E. Coli	NP_418620.1	9		1	0	0	0
E. Coli	NP_416876.1	19		1	0	0	0

K. pneumoniae	YP_005228912.1	13	11			1	0	0	0
K. pneumoniae	YP_005226061.1	11				1	0	0	0
E. Coli	NP_417301.4	17				1	0	0	0
S. enterica	NP_461915.1	18				1	0	0	0
K. pneumoniae	YP_005228596.1	23				1	0	0	0
S. enterica	NP_459129.1	14				1	0	0	0
S. enterica	NP_459843.1	12				1	0	0	0
E. Coli	NP_415445.1	12				1	0	0	0
S. enterica	NP_459970.1	12				1	0	0	0
K. pneumoniae	YP_005226133.1	12				1	0	0	0
S. enterica	NP_460408.1	14				1	0	0	0
K. pneumoniae	YP_005227258.1	16				1	0	0	0
E. Coli	NP_417312.1	12				1	0	0	0
S. enterica	NP_461926.1	14	1			1	0	0	0
K. pneumoniae	YP_005228608.1	17				1	0	0	0
E. Coli	NP_418564.2	6				1	0	0	0
S. enterica	NP_463191.3	6				1	0	0	0
K. pneumoniae	YP_005224676.1	6	11			1	0	0	0
S. enterica	NP_463239.1	13				1	0	0	0
E. Coli	NP_415583.4	14				1	0	0	0
S. enterica	NP_460137.1	14				1	0	0	0
K. pneumoniae	YP_005226254.1	14				1	0	0	0
E. Coli	NP_415709.2	11				1	0	0	0
S. enterica	NP_460757.1	11				1	0	0	0
E. Coli	NP_417314.1	8				1	0	0	0
S. enterica	NP_461928.1	8				1	0	0	0
K. pneumoniae	YP_005228611.1	9				1	0	0	0
E. Coli	NP_417006.2	1				1	0	0	0
K. pneumoniae	YP_005228181.1	2				1	0	0	0
E. Coli	NP_417631.2	11	17	19	12	1	0	0	0
S. enterica	NP_462193.3	11	16	12		1	0	0	0
K. pneumoniae	YP_005229007.1	11	21	48		1	0	0	0
E. Coli	NP_416581.1	11	15			1	0	0	0
S. enterica	NP_461074.1	15				1	0	0	0
E. Coli	NP_414660.1	11				1	0	0	0
S. enterica	NP_459163.1	11				1	0	0	0
E. Coli	NP_414931.1	17				1	0	0	0
S. enterica	NP_459390.1	17	1			1	0	0	0
K. pneumoniae	YP_005225376.1	14	28	13		1	0	0	0
E. Coli	NP_416146.1	15	21			1	0	0	0
S. enterica	NP_460420.1	15	14			1	0	0	0
K. pneumoniae	YP_005227244.1	22				1	0	0	0
E. Coli	NP_415167.1	11				1	0	0	0
S. enterica	NP_459631.1	11				1	0	0	0
K. pneumoniae	YP_005225805.1	1				1	0	0	0
E. Coli	NP_414597.1	12				1	0	0	0
E. Coli	NP_414655.1	2				1	0	0	0

S. enterica	NP_459156.1	15					1	0	0	0
E. Coli	NP_415622.1	9					1	0	0	0
E. Coli	NP_415619.1	13					1	0	0	0
S. enterica	NP_460173.1	13					1	0	0	0
K. pneumoniae	YP_005226278.1	13					1	0	0	0
E. Coli	NP_417082.1	9					1	0	0	0
K. pneumoniae	YP_005228282.1	14					1	0	0	0
K. pneumoniae	YP_005225997.1	16					1	0	0	0
E. Coli	NP_414659.1	21					1	0	0	0
S. enterica	NP_459162.2	14	23	2	14		1	0	0	0
K. pneumoniae	YP_005225143.1	15	21	12	14	14	1	0	0	0
E. Coli	NP_414870.1	1					1	0	0	0
S. enterica	NP_462243.1	1					1	0	0	0
S. enterica	NP_462663.1	11					1	0	0	0
S. enterica	NP_459908.1	1					1	0	0	0
S. enterica	NP_461107.1	14	11				1	0	0	0
E. Coli	NP_414556.1	1	12				1	0	0	0
S. enterica	NP_459018.1	13	12				1	0	0	0
K. pneumoniae	YP_005225025.1	11	12				1	0	0	0
S. enterica	NP_462473.1	11					1	0	0	0
E. Coli	NP_416579.1	11	11				1	0	0	0
S. enterica	NP_461072.1	12	11				1	0	0	0
K. pneumoniae	YP_005227887.1	11	11				1	0	0	0
K. pneumoniae	YP_005225207.1	16					1	0	0	0
S. enterica	NP_462511.1	11					1	0	0	0
K. pneumoniae	YP_005229330.1	11					1	0	0	0
K. pneumoniae	YP_005228276.1	23					1	0	0	0
E. Coli	NP_415990.5	16					1	0	0	0
S. enterica	NP_460530.3	13					1	0	0	0
K. pneumoniae	YP_005227138.1	14					1	0	0	0
E. Coli	NP_418454.1	14					1	0	0	0
S. enterica	NP_463091.1	15					1	0	0	0
E. Coli	NP_416252.1	11					1	0	0	0
S. enterica	NP_460278.1	11					1	0	0	0
K. pneumoniae	YP_005226428.1	11					1	0	0	0
S. enterica	NP_459495.1	19					1	0	0	0
E. Coli	NP_416649.1	16					1	0	0	0
S. enterica	NP_461129.1	14					1	0	0	0
K. pneumoniae	YP_005227948.1	14					1	0	0	0
K. pneumoniae	YP_005228958.1	16					1	0	0	0
S. enterica	NP_462756.1	9					1	0	0	0
K. pneumoniae	YP_005229600.1	9					1	0	0	0
E. Coli	NP_416042.2	11					1	0	0	0
S. enterica	NP_460484.1	11					1	0	0	0
K. pneumoniae	YP_005226865.1	11					1	0	0	0
E. Coli	NP_414983.1	12					1	0	0	0
K. pneumoniae	YP_005225454.1	12					1	0	0	0

K. pneumoniae	YP_005227180.1	16				1	0	0	0
K. pneumoniae	YP_005228035.1	17				1	0	0	0
S. enterica	NP_459174.1	11				1	0	0	0
K. pneumoniae	YP_005225158.1	11				1	0	0	0
K. pneumoniae	YP_005229098.1	18				1	0	0	0
K. pneumoniae	YP_005229287.1	1				1	0	0	0
E. Coli	NP_415571.1	11				1	0	0	0
S. enterica	NP_460125.1	11	12			1	0	0	0
K. pneumoniae	YP_005226243.1	11				1	0	0	0
E. Coli	NP_416827.4	9	13			1	0	0	0
S. enterica	NP_461321.3	9				1	0	0	0
K. pneumoniae	YP_005228083.1	9				1	0	0	0
E. Coli	NP_417222.1	32	17			1	0	0	0
S. enterica	NP_461846.1	14				1	0	0	0
K. pneumoniae	YP_005228479.1	35				1	0	0	0
K. pneumoniae	YP_005225063.1	8				1	0	0	0
E. Coli	NP_415428.1	11				1	0	0	0
E. Coli	NP_418220.1	12				1	0	0	0
S. enterica	NP_462796.1	12				1	0	0	0
K. pneumoniae	YP_005224418.1	12				1	0	0	0
E. Coli	NP_414609.1	17	18	1		1	0	0	0
S. enterica	NP_459112.1	19				1	0	0	0
K. pneumoniae	YP_005225080.1	11	19	12		1	0	0	0
E. Coli	NP_415634.1	2				1	0	0	0
S. enterica	NP_460187.3	2				1	0	0	0
K. pneumoniae	YP_005226297.1	13				1	0	0	0
E. Coli	NP_416867.1	8				1	0	0	0
S. enterica	NP_462703.1	8				1	0	0	0
K. pneumoniae	YP_005229554.1	8	11			1	0	0	0
S. enterica	NP_462482.3	13				1	0	0	0
E. Coli	NP_416360.1	15	15			1	0	0	0
S. enterica	NP_460837.1	15	16			1	0	0	0
K. pneumoniae	YP_005224704.1	14				1	0	0	0
K. pneumoniae	YP_005224815.1	13				1	0	0	0
E. Coli	NP_417913.1	9	2	14	11	1	0	0	0
S. enterica	NP_462463.1	2	14	11		1	0	0	0
K. pneumoniae	YP_005229271.1	2	14	11		1	0	0	0
K. pneumoniae	YP_005229235.1	16				1	0	0	0
E. Coli	NP_416630.1	18				1	0	0	0
S. enterica	NP_461104.1	18				1	0	0	0
K. pneumoniae	YP_005227926.1	18				1	0	0	0
E. Coli	NP_417763.1	1				1	0	0	0
S. enterica	NP_462328.1	1				1	0	0	0
K. pneumoniae	YP_005229152.1	1				1	0	0	0
E. Coli	NP_416578.2	12				1	0	0	0
S. enterica	NP_461071.1	11				1	0	0	0
K. pneumoniae	YP_005227886.1	17				1	0	0	0

E. Coli	NP_418679.1	11				1	0	0	0
S. enterica	NP_463335.1	11				1	0	0	0
K. pneumoniae	YP_005224816.1	11				1	0	0	0
K. pneumoniae	YP_005224367.1	13				1	0	0	0
K. pneumoniae	YP_005229282.1	12				1	0	0	0
K. pneumoniae	YP_005227735.1	24				1	0	0	0
E. Coli	NP_415995.4	12				1	0	0	0
S. enterica	NP_460526.1	12				1	0	0	0
K. pneumoniae	YP_005227123.1	12				1	0	0	0
K. pneumoniae	YP_005228512.1	11				1	0	0	0
E. Coli	NP_415961.1	12				1	0	0	0
E. Coli	NP_415753.1	15				1	0	0	0
S. enterica	NP_460710.1	15				1	0	0	0
K. pneumoniae	YP_005227498.1	2				1	0	0	0
K. pneumoniae	YP_005228934.1	11				1	0	0	0
K. pneumoniae	YP_005227728.1	12				1	0	0	0
E. Coli	NP_415314.1	14				1	0	0	0
K. pneumoniae	YP_005228067.1	12				1	0	0	0
K. pneumoniae	YP_005224428.1	16				1	0	0	0
S. enterica	NP_460705.3	13				1	0	0	0
S. enterica	NP_462735.1	12				1	0	0	0
K. pneumoniae	YP_005229575.1	12				1	0	0	0
E. Coli	NP_415056.1	14				1	0	0	0
S. enterica	NP_459529.1	14				1	0	0	0
E. Coli	NP_418247.1	18	13			1	0	0	0
S. enterica	NP_462822.1	19	12			1	0	0	0
K. pneumoniae	YP_005224446.1	18	17			1	0	0	0
S. enterica	NP_459440.1	13				1	0	0	0
K. pneumoniae	YP_005225435.1	17				1	0	0	0
E. Coli	NP_414697.1	13	12	18	11	1	0	0	0
K. pneumoniae	YP_005225204.1	14	13			1	0	0	0
E. Coli	NP_418573.1	12				1	0	0	0
S. enterica	NP_460724.3	9				1	0	0	0
E. Coli	NP_418116.1	1				1	0	0	0
S. enterica	NP_462664.1	1	11			1	0	0	0
K. pneumoniae	YP_005229514.1	1				1	0	0	0
S. enterica	NP_463446.1	13				1	0	0	0
K. pneumoniae	YP_005224693.1	12				1	0	0	0
S. enterica	NP_463058.1	9				1	0	0	0
K. pneumoniae	YP_005224543.1	11				1	0	0	0
S. enterica	NP_462300.1	19				1	0	0	0
K. pneumoniae	YP_005229126.1	14				1	0	0	0
E. Coli	NP_416330.1	12				1	0	0	0
S. enterica	NP_460784.2	12				1	0	0	0
S. enterica	NP_460601.1	1				1	0	0	0
K. pneumoniae	YP_005226170.1	13				1	0	0	0
E. Coli	NP_415059.1	16				1	0	0	0

S. enterica	NP_459532.1	18						1	0	0	0
K. pneumoniae	YP_005225531.1	16						1	0	0	0
E. Coli	NP_416653.1	26						1	0	0	0
S. enterica	NP_461133.1	2						1	0	0	0
K. pneumoniae	YP_005227949.1	2						1	0	0	0
S. enterica	NP_462971.1	18						1	0	0	0
K. pneumoniae	YP_005224377.1	18						1	0	0	0
E. Coli	NP_417662.1	11						1	0	0	0
S. enterica	NP_462223.1	11						1	0	0	0
K. pneumoniae	YP_005229044.1	11						1	0	0	0
E. Coli	NP_415602.1	3	14	12	17	16	44	1	0	0	0
S. enterica	NP_460156.1	17	11	11				1	0	0	0
K. pneumoniae	YP_005226262.1	17	32	15	22	2		1	0	0	0
E. Coli	NP_416056.1	1						1	0	0	0
S. enterica	NP_460472.1	1						1	0	0	0
K. pneumoniae	YP_005226803.1	6	1					1	0	0	0
E. Coli	NP_416467.1	12						1	0	0	0
E. Coli	NP_415741.1	13						1	0	0	0
S. enterica	NP_460721.1	13						1	0	0	0
S. enterica	NP_459248.1	11						1	0	0	0
K. pneumoniae	YP_005224399.1	11						1	0	0	0
E. Coli	NP_415266.1	11						1	0	0	0
S. enterica	NP_459731.1	11						1	0	0	0
K. pneumoniae	YP_005225880.1	11						1	0	0	0
K. pneumoniae	YP_005229418.1	11						1	0	0	0
E. Coli	NP_417853.1	19						1	0	0	0
S. enterica	NP_462394.1	18						1	0	0	0
K. pneumoniae	YP_005229216.1	12						1	0	0	0
E. Coli	NP_414555.1	12						1	0	0	0
S. enterica	NP_459017.1	9						1	0	0	0
K. pneumoniae	YP_005225024.1	9						1	0	0	0
E. Coli	NP_415721.1	11						1	0	0	0
S. enterica	NP_460740.1	11						1	0	0	0
K. pneumoniae	YP_005225109.1	9						1	0	0	0
S. enterica	NP_462956.1	11						1	0	0	0
K. pneumoniae	YP_005228939.1	14						1	0	0	0
K. pneumoniae	YP_005226502.1	8	15					1	0	0	0
K. pneumoniae	YP_005227506.1	12						1	0	0	0
S. enterica	NP_463249.1	15						1	0	0	0
S. enterica	NP_462779.1	11						1	0	0	0
E. Coli	NP_414964.1	12						1	0	0	0
S. enterica	NP_459437.1	12						1	0	0	0
E. Coli	NP_417055.4	19						1	0	0	0
S. enterica	NP_461504.1	19						1	0	0	0
E. Coli	NP_415316.1	16	12					1	0	0	0
S. enterica	NP_459796.1	16	12					1	0	0	0
K. pneumoniae	YP_005225950.1	16	12					1	0	0	0

S. enterica	NP_460035.1	11							1	0	0	0
E. Coli	NP_417904.1	8							1	0	0	0
K. pneumoniae	YP_005225229.1	12							1	0	0	0
K. pneumoniae	YP_005228241.1	18							1	0	0	0
S. enterica	NP_461954.1	11							1	0	0	0
K. pneumoniae	YP_005228683.1	13							1	0	0	0
S. enterica	NP_462777.3	11							1	0	0	0
K. pneumoniae	YP_005224304.1	1							1	0	0	0
K. pneumoniae	YP_005225877.1	12							1	0	0	0
E. Coli	NP_415935.2	11							1	0	0	0
E. Coli	NP_417090.1	11							1	0	0	0
S. enterica	NP_461597.1	11							1	0	0	0
K. pneumoniae	YP_005228407.1	12							1	0	0	0
E. Coli	NP_418319.2	16							1	0	0	0
S. enterica	NP_462904.3	16							1	0	0	0
S. enterica	NP_461206.1	12							1	0	0	0
S. enterica	NP_461353.1	11							1	0	0	0
E. Coli	NP_417921.1	11	21	14					1	0	0	0
S. enterica	NP_462472.1	11	15						1	0	0	0
K. pneumoniae	YP_005229284.1	13	23						1	0	0	0
E. Coli	NP_418110.1	15	13						1	0	0	0
S. enterica	NP_462646.1	13	16						1	0	0	0
K. pneumoniae	YP_005229478.1	13							1	0	0	0
K. pneumoniae	YP_005225453.1	12							1	0	0	0
E. Coli	NP_417808.1	47							1	0	0	0
S. enterica	NP_462359.1	47							1	0	0	0
K. pneumoniae	YP_005229184.1	46							1	0	0	0
K. pneumoniae	YP_005228767.1	12							1	0	0	0
S. enterica	NP_459630.1	45							1	0	0	0
K. pneumoniae	YP_005225804.1	21	25						1	0	0	0
E. Coli	NP_416143.2	2							1	0	0	0
S. enterica	NP_460423.1	16							1	0	0	0
K. pneumoniae	YP_005227241.1	19	12						1	0	0	0
S. enterica	NP_460325.1	13							1	0	0	0
S. enterica	NP_460780.1	11							1	0	0	0
S. enterica	NP_459059.1	12	9	1					0	0	0	1
S. enterica	NP_459747.1	12	9	2					0	0	0	1
S. enterica	NP_462261.1	12	9	1					0	0	0	1
K. pneumoniae	YP_005225040.1	9	12	9	2				0	0	0	1
E. Coli	NP_415890.2	69	25	32	55	1	3	25	0	0	0	1
S. enterica	NP_461524.1	27	28	28	28	51	7		0	0	0	1
S. enterica	NP_460024.1	27	28	23	82	11			0	0	0	1
S. enterica	NP_459903.1	41	26	23	77	14			0	0	0	1
K. pneumoniae	YP_005225711.1	12							0	0	0	1
S. enterica	NP_461124.1	14							0	0	0	1
S. enterica	NP_459746.1	15							0	0	0	1
S. enterica	NP_462263.1	15							0	0	0	1

K. pneumoniae	YP_005225042.1	16				0	0	0	1
K. pneumoniae	YP_005227205.1	18				0	0	0	1
E. Coli	NP_417446.4	11				0	0	0	1
K. pneumoniae	YP_005225176.1	18				0	0	0	1
K. pneumoniae	YP_005224356.1	1				0	0	0	1
S. enterica	NP_462712.1	9				0	0	0	1
K. pneumoniae	YP_005225041.1	29				0	0	0	1
E. Coli	NP_416669.1	16				0	0	0	1
E. Coli	NP_416003.1	2				0	0	1	0
E. Coli	NP_418721.1	12				0	0	1	0
S. enterica	NP_460576.1	11				0	0	1	0
E. Coli	NP_416216.5	14				0	0	1	0
K. pneumoniae	YP_005227407.1	11				0	0	1	0
S. enterica	NP_461358.1	11				0	0	1	0
E. Coli	NP_414875.1	9	11			0	0	1	0
K. pneumoniae	YP_005228104.1	16	11	17	1	0	0	1	0
S. enterica	NP_460533.1	26				0	0	1	0
K. pneumoniae	YP_005227147.1	26	15			0	0	1	0
S. enterica	NP_460148.1	14				0	0	1	0
E. Coli	NP_416453.1	11				0	0	1	0
S. enterica	NP_460927.1	14	1	11		0	0	1	0
E. Coli	NP_418540.1	15				0	0	1	0
S. enterica	NP_461003.1	11				0	0	1	0
K. pneumoniae	YP_005228586.1	11				0	0	1	0
E. Coli	NP_415854.4	15				0	0	1	0
K. pneumoniae	YP_005226742.1	13				0	0	1	0
K. pneumoniae	YP_005225697.1	19				0	0	1	0
S. enterica	NP_461284.3	16				0	0	1	0
S. enterica	NP_461625.1	15	12	18	13	0	0	1	0
K. pneumoniae	YP_005226048.1	13				0	0	1	0
E. Coli	NP_416289.1	11				0	0	1	0
K. pneumoniae	YP_005226387.1	11				0	0	1	0
E. Coli	NP_417451.1	15				0	0	1	0
S. enterica	NP_460988.1	9				0	0	1	0
K. pneumoniae	YP_005228571.1	9				0	0	1	0
S. enterica	NP_461287.1	1				0	0	1	0
S. enterica	NP_459160.1	9				0	0	1	0
E. Coli	NP_416185.1	12				0	0	1	0
S. enterica	NP_461008.1	11	12			0	0	1	0
E. Coli	NP_415513.2	14				0	0	1	0
K. pneumoniae	YP_005227715.1	16				0	0	1	0
K. pneumoniae	YP_005228459.1	12				0	0	1	0
E. Coli	NP_416611.1	11				0	0	1	0
E. Coli	NP_415182.1	14				0	0	1	0
K. pneumoniae	YP_005229687.1	12				0	0	1	0
S. enterica	NP_463428.1	11				0	0	1	0
S. enterica	NP_460987.1	7				0	0	1	0

S. enterica	NP_459200.1	1		0	0	1	0
S. enterica	NP_459511.1	12		0	0	1	0
S. enterica	NP_460585.2	18		0	0	1	0
E. Coli	NP_418724.1	15		0	0	1	0
S. enterica	NP_460573.1	13		0	0	1	0
S. enterica	NP_460825.1	12		0	0	1	0
K. pneumoniae	YP_005226868.1	21		0	0	1	0
S. enterica	NP_462064.1	11		0	0	1	0
S. enterica	NP_461647.1	11	15	0	0	1	0
S. enterica	NP_463304.1	15		0	0	1	0
K. pneumoniae	YP_005224781.1	15		0	0	1	0
S. enterica	NP_463386.1	11		0	0	1	0
S. enterica	NP_459033.1	12		0	0	1	0
E. Coli	NP_416769.1	14		0	0	1	0
S. enterica	NP_461253.3	14		0	0	1	0
E. Coli	NP_416554.1	12		0	0	1	0
E. Coli	NP_418734.1	16		0	0	1	0
K. pneumoniae	YP_005228655.1	11		0	0	1	0
S. enterica	NP_460749.1	15		0	0	1	0
E. Coli	NP_415598.3	1	1	0	0	1	0
S. enterica	NP_460152.1	1	1	0	0	1	0
E. Coli	NP_416266.1	11		0	0	1	0
K. pneumoniae	YP_005226414.1	2		0	0	1	0
E. Coli	NP_416457.4	12		0	0	1	0
S. enterica	NP_460931.3	13		0	0	1	0
E. Coli	NP_417471.1	11	16	0	0	1	0
S. enterica	NP_462065.1	11	19	0	0	1	0
E. Coli	NP_418023.1	1		0	0	1	0
S. enterica	NP_459581.1	9		0	0	1	0
S. enterica	NP_461864.1	11	18	0	0	1	0
E. Coli	NP_416839.1	15		0	0	1	0
E. Coli	NP_415525.2	11		0	0	1	0
K. pneumoniae	YP_005225328.1	1		0	0	1	0
E. Coli	NP_416174.1	15		0	0	1	0
K. pneumoniae	YP_005227277.1	15		0	0	1	0
S. enterica	NP_462571.1	11		0	0	1	0
S. enterica	NP_490585.1	14		0	0	1	0
S. enterica	NP_460505.1	13		0	0	1	0
E. Coli	NP_417935.1	13		0	0	1	0
K. pneumoniae	YP_005225330.1	12		0	0	1	0
K. pneumoniae	YP_005228424.1	13	14	0	0	1	0
S. enterica	NP_459928.1	11		0	0	1	0
S. enterica	NP_459974.1	16		0	0	1	0
S. enterica	NP_461880.1	11		0	0	1	0
K. pneumoniae	YP_005228506.1	1		0	0	1	0
K. pneumoniae	YP_005229761.1	12		0	0	1	0
K. pneumoniae	YP_005229756.1	12		0	0	1	0

K. pneumoniae	YP_005225185.1	15		0	0	1	0
K. pneumoniae	YP_005228569.1	22		0	0	1	0
S. enterica	NP_460878.1	13		0	0	1	0
S. enterica	NP_459617.1	11		0	0	1	0
K. pneumoniae	YP_005226187.1	14		0	0	1	0
K. pneumoniae	YP_005228579.1	9		0	0	1	0
S. enterica	NP_462572.1	19		0	0	1	0
E. Coli	NP_416773.1	15		0	0	1	0
S. enterica	NP_461257.1	31		0	0	1	0
E. Coli	NP_418025.1	12		0	0	1	0
K. pneumoniae	YP_005229391.1	1		0	0	1	0
S. enterica	NP_459271.1	1		0	0	1	0
E. Coli	NP_417829.2	12		0	0	1	0
E. Coli	NP_416755.1	13		0	0	1	0
E. Coli	NP_416459.1	14	13	0	0	1	0
S. enterica	NP_460933.1	13		0	0	1	0
S. enterica	NP_459064.1	12		0	0	1	0
K. pneumoniae	YP_005225045.1	15		0	0	1	0
K. pneumoniae	YP_005226050.1	13		0	0	1	0
K. pneumoniae	YP_005225179.1	1		0	0	1	0
E. Coli	NP_416460.1	21		0	0	1	0
S. enterica	NP_460934.1	13		0	0	1	0
S. enterica	NP_461198.1	18		0	0	1	0
E. Coli	NP_418128.1	9		0	0	1	0
S. enterica	NP_462696.1	9		0	0	1	0
S. enterica	NP_462131.1	15		0	0	1	0
K. pneumoniae	YP_005225324.1	12		0	0	1	0
K. pneumoniae	YP_005229306.1	21	15	0	0	1	0
K. pneumoniae	YP_005226003.1	16		0	0	1	0
K. pneumoniae	YP_005229696.1	11		0	0	1	0
E. Coli	NP_416434.1	17		0	0	1	0
K. pneumoniae	YP_005228653.1	11		0	0	1	0
S. enterica	NP_461087.1	11		0	0	1	0
E. Coli	NP_415454.4	2		0	0	1	0
K. pneumoniae	YP_005226150.1	11	15	0	0	1	0
S. enterica	NP_490567.1	13		0	0	1	0
K. pneumoniae	YP_005229715.1	15	2	0	0	1	0
K. pneumoniae	YP_005225186.1	11		0	0	1	0
S. enterica	NP_460978.1	14		0	0	1	0
K. pneumoniae	YP_005228562.1	11		0	0	1	0
E. Coli	NP_415852.2	1		0	0	1	0
E. Coli	NP_416270.1	11		0	0	1	0
S. enterica	NP_461861.1	1		0	0	1	0
S. enterica	NP_460924.1	1		0	0	1	0
K. pneumoniae	YP_005227107.1	11		0	0	1	0
E. Coli	NP_415959.1	12		0	0	1	0
K. pneumoniae	YP_005227201.1	1		0	0	1	0

E. Coli	NP_418775.1	18			0	0	1	0
S. enterica	NP_463392.1	15	18		0	0	1	0
K. pneumoniae	YP_005229695.1	1			0	0	1	0
E. Coli	NP_415514.1	1			0	0	1	0
E. Coli	NP_416105.4	11			0	0	1	0
E. Coli	NP_417689.4	12	9		0	0	1	0
S. enterica	NP_462246.1	9			0	0	1	0
K. pneumoniae	YP_005225349.1	14			0	0	1	0
K. pneumoniae	YP_005228929.1	14	11		0	0	1	0
K. pneumoniae	YP_005228178.1	6			0	0	1	0
E. Coli	NP_418651.3	13			0	0	1	0
K. pneumoniae	YP_005224767.1	1	12		0	0	1	0
S. enterica	NP_460048.1	11			0	0	1	0
S. enterica	NP_462235.1	12			0	0	1	0
S. enterica	NP_461338.1	12			0	0	1	0
K. pneumoniae	YP_005226963.1	12			0	0	1	0
K. pneumoniae	YP_005226859.1	12			0	0	1	0
S. enterica	NP_460433.1	12			0	0	1	0
K. pneumoniae	YP_005226676.1	11	9	13	0	0	1	0
S. enterica	NP_462041.1	17			0	0	1	0
K. pneumoniae	YP_005225351.1	15			0	0	1	0
K. pneumoniae	YP_005228238.1	13	9		0	0	1	0
S. enterica	NP_490498.3	14			0	0	1	0
E. Coli	NP_415150.1	11			0	0	1	0
S. enterica	NP_459062.1	18			0	0	1	0
K. pneumoniae	YP_005225043.1	16			0	0	1	0
E. Coli	NP_418117.1	12			0	0	1	0
K. pneumoniae	YP_005229496.1	13			0	0	1	0
S. enterica	NP_463236.1	13			0	0	1	0
E. Coli	NP_418384.1	13			0	0	1	0
S. enterica	NP_462992.1	14			0	0	1	0
E. Coli	NP_415560.1	15			0	0	1	0
S. enterica	NP_460115.1	13			0	0	1	0
K. pneumoniae	YP_005224662.1	12			0	0	1	0
K. pneumoniae	YP_005224821.1	13			0	0	1	0
K. pneumoniae	YP_005224882.1	12			0	0	1	0
S. enterica	NP_459507.1	12			0	0	1	0
S. enterica	NP_459028.1	11			0	0	1	0
E. Coli	NP_414583.2	13			0	0	1	0
E. Coli	NP_415326.1	13			0	0	1	0
E. Coli	NP_415464.4	14			0	0	1	0
S. enterica	NP_462922.1	4	1		0	0	1	0
K. pneumoniae	YP_005224352.1	4	1		0	0	1	0
K. pneumoniae	YP_005228700.1	16			0	0	1	0
S. enterica	NP_459279.1	12			0	0	1	0
E. Coli	NP_415620.1	13			0	0	1	0
S. enterica	NP_460174.1	14			0	0	1	0

K. pneumoniae	YP_005228667.1	1	8			0	0	1	0
K. pneumoniae	YP_005228550.1	1				0	0	1	0
E. Coli	NP_416269.4	16	16	7	13	0	0	1	0
K. pneumoniae	YP_005226411.1	11	22	16		0	0	1	0
K. pneumoniae	YP_005225183.1	11				0	0	1	0
E. Coli	NP_415179.1	1				0	0	1	0
K. pneumoniae	YP_005228453.1	19				0	0	1	0
E. Coli	NP_418345.2	15				0	0	1	0
E. Coli	NP_415827.1	14				0	0	1	0
K. pneumoniae	YP_005228206.1	11				0	0	1	0
S. enterica	NP_462921.1	14				0	0	1	0
E. Coli	NP_416553.1	1				0	0	1	0
S. enterica	NP_461050.3	1				0	0	1	0
S. enterica	NP_459376.1	14				0	0	1	0
K. pneumoniae	YP_005225362.1	13				0	0	1	0
E. Coli	NP_418498.1	12				0	0	1	0
S. enterica	NP_463146.1	13	14	12		0	0	1	0
K. pneumoniae	YP_005228419.1	13				0	0	1	0
E. Coli	NP_417861.1	13				0	0	1	0
S. enterica	NP_462402.1	11	15			0	0	1	0
S. enterica	NP_459161.1	1	1			0	0	1	0
K. pneumoniae	YP_005227182.1	16				0	0	1	0
K. pneumoniae	YP_005225889.1	19	9			0	0	1	0
E. Coli	NP_415087.1	11				0	0	1	0
S. enterica	NP_459027.1	16				0	0	1	0
E. Coli	NP_416394.1	13				0	0	1	0
E. Coli	NP_418215.1	13				0	0	1	0
S. enterica	NP_462791.1	13				0	0	1	0
E. Coli	NP_417037.1	6	23			0	0	1	0
S. enterica	NP_460249.1	12				0	0	1	0
S. enterica	NP_460347.1	13				0	0	1	0
K. pneumoniae	YP_005226941.1	1				0	0	1	0
E. Coli	NP_414825.1	12	11			0	0	1	0
K. pneumoniae	YP_005225329.1	12				0	0	1	0
K. pneumoniae	YP_005227202.1	18				0	0	1	0
E. Coli	NP_415844.1	11				0	0	1	0
E. Coli	NP_415912.1	13				0	0	1	0
E. Coli	NP_417783.1	15				0	0	1	0
E. Coli	NP_416571.1	15				0	0	1	0
S. enterica	NP_461068.1	12				0	0	1	0
S. enterica	NP_462248.1	4				0	0	1	0
E. Coli	NP_418499.1	12				0	0	1	0
S. enterica	NP_459705.1	11				0	0	1	0
K. pneumoniae	YP_005227399.1	16				0	0	1	0
S. enterica	NP_460105.1	12				0	0	1	0
S. enterica	NP_460995.1	12				0	0	1	0
E. Coli	NP_416901.1	11				0	0	1	0

S. enterica	NP_461356.1	11		0	0	1	0
K. pneumoniae	YP_005229305.1	13		0	0	1	0
K. pneumoniae	YP_005229685.1	11	15	0	0	1	0
K. pneumoniae	YP_005224887.1	14		0	0	1	0
K. pneumoniae	YP_005226444.1	11		0	0	1	0
E. Coli	NP_416448.1	11		0	0	1	0
E. Coli	NP_415491.1	21		0	0	1	0
S. enterica	NP_460742.1	12		0	0	1	0
E. Coli	NP_415494.1	15		0	0	1	0
S. enterica	NP_460745.1	14		0	0	1	0
S. enterica	NP_463182.1	9		0	0	1	0
K. pneumoniae	YP_005228835.1	9		0	0	1	0
K. pneumoniae	YP_005229714.1	13		0	0	1	0
E. Coli	NP_417523.1	14	13	0	0	1	0
S. enterica	NP_462114.1	13		0	0	1	0
E. Coli	NP_415597.1	1		0	0	1	0
S. enterica	NP_460151.1	1		0	0	1	0
E. Coli	NP_416400.1	14	18	0	0	1	0
S. enterica	NP_460876.1	18		0	0	1	0
E. Coli	NP_418176.1	1		0	0	1	0
S. enterica	NP_460998.1	13		0	0	1	0
K. pneumoniae	YP_005228581.1	13		0	0	1	0
S. enterica	NP_463137.1	12		0	0	1	0
K. pneumoniae	YP_005224609.1	12		0	0	1	0
K. pneumoniae	YP_005228656.1	1		0	0	1	0
K. pneumoniae	YP_005229719.1	18		0	0	1	0
S. enterica	NP_459749.1	17		0	0	1	0
K. pneumoniae	YP_005225633.1	23	7	0	0	1	0
S. enterica	NP_461120.1	11		0	0	1	0
E. Coli	NP_416340.1	12		0	0	1	0
S. enterica	NP_460796.1	12		0	0	1	0
E. Coli	NP_417220.1	13		0	0	1	0
K. pneumoniae	YP_005226816.1	18	11	0	0	1	0
E. Coli	NP_415593.1	13		0	0	1	0
E. Coli	NP_414887.2	12		0	0	1	0
K. pneumoniae	YP_005227398.1	17		0	0	1	0
K. pneumoniae	YP_005224864.1	11		0	0	1	0
K. pneumoniae	YP_005228927.1	14		0	0	1	0
K. pneumoniae	YP_005226490.1	18	11	0	0	1	0
K. pneumoniae	YP_005224634.1	11		0	0	1	0
S. enterica	NP_459346.1	15		0	0	1	0
K. pneumoniae	YP_005225694.1	15	13	0	0	1	0
S. enterica	NP_459527.1	11		0	0	1	0
K. pneumoniae	YP_005225684.1	11		0	0	1	0
E. Coli	NP_416214.1	15		0	0	1	0
S. enterica	NP_460318.1	15	16	0	0	1	0
E. Coli	NP_416708.1	12		0	0	1	0

E. Coli	NP_417959.4	12			0	0	1	0
K. pneumoniae	YP_005228672.1	15			0	0	1	0
K. pneumoniae	YP_005226412.1	7			0	0	1	0
E. Coli	NP_417791.1	17			0	0	1	0
K. pneumoniae	YP_005225180.1	11			0	0	1	0
S. enterica	NP_461738.1	18	14		0	0	1	0
K. pneumoniae	YP_005227236.1	11	11		0	0	1	0
S. enterica	NP_462661.1	23			0	0	1	0
K. pneumoniae	YP_005227305.1	33			0	0	1	0
E. Coli	NP_417251.2	11	13	11	0	0	1	0
S. enterica	NP_460946.1	14	8		0	0	1	0
S. enterica	NP_461642.1	12			0	0	1	0
K. pneumoniae	YP_005226029.1	12			0	0	1	0
K. pneumoniae	YP_005229319.1	1			0	0	1	0
S. enterica	NP_490588.1	33			0	0	1	0
K. pneumoniae	YP_005229688.1	23			0	0	1	0
S. enterica	NP_459524.1	11			0	0	1	0
K. pneumoniae	YP_005228625.1	11			0	0	1	0
E. Coli	NP_415047.1	14			0	0	1	0
S. enterica	NP_459520.1	12			0	0	1	0
K. pneumoniae	YP_005228560.1	1			0	0	1	0
E. Coli	NP_414580.1	1			0	0	1	0
S. enterica	NP_461337.1	11			0	0	1	0
E. Coli	NP_416231.1	11			0	0	1	0
S. enterica	NP_460302.1	11			0	0	1	0
S. enterica	NP_461833.1	12			0	0	1	0
K. pneumoniae	YP_005228475.1	12			0	0	1	0
K. pneumoniae	YP_005228384.1	11			0	0	1	0
S. enterica	NP_460086.1	12			0	0	1	0
K. pneumoniae	YP_005228699.1	13			0	0	1	0
K. pneumoniae	YP_005228465.1	17	12	1	0	0	1	0
S. enterica	NP_462581.1	11			0	0	1	0
S. enterica	NP_460748.1	8			0	0	1	0
S. enterica	NP_459287.1	18			0	0	1	0
E. Coli	NP_415575.1	11			0	0	1	0
S. enterica	NP_460129.1	6			0	0	1	0
S. enterica	NP_453702.1	12			0	0	1	0
E. Coli	NP_418506.1	12			0	0	1	0
K. pneumoniae	YP_005228666.1	15			0	0	1	0
S. enterica	NP_463147.1	1			0	0	1	0
K. pneumoniae	YP_005224826.1	16			0	0	1	0
E. Coli	NP_416560.1	11			0	0	1	0
K. pneumoniae	YP_005227406.1	12			0	0	1	0
K. pneumoniae	YP_005228476.1	18			0	0	1	0
E. Coli	NP_416393.1	11			0	0	1	0
S. enterica	NP_460870.1	11	11		0	0	1	0
S. enterica	NP_461024.1	12			0	0	1	0

S. enterica	NP_459422.1	18	0	0	1	0
S. enterica	NP_463385.1	11	0	0	1	0
