# Supplementary material for: Higher frequency of prokaryotic low complexity regions in core and orthologous genes
Source: Front Bioinform. 2025 Nov 27;5:1673480. doi: 10.3389/fbinf.2025.1673480 (PMC12695832; doi:10.3389/fbinf.2025.1673480)
Supplement: Supplementary file 2 [file DataSheet1.pdf]

Supplementary Material

Table S1 Composition of each random set of strains in *E. coli*, *S. enterica*, and *K. pneumoniae*. Assembly identifiers from NCBI are listed.

	**Dataset 1**	**Dataset 2**	**Dataset 3**
Escherichia coli	ASM435840v1	ASM301805v1	ASM886v2
	ASM522188v1	ASM301813v1	ASM172112v1
	ASM1199608v1	ASM301845v1	ASM301791v1
	ASM1335736v1	ASM301855v1	ASM301803v1
	ASM1405844v2	ASM301857v1	ASM2792574v1
	ASM2430068v1	ASM301879v1	ASM2792576v1
	ASM2599567v1	ASM301889v1	ASM2792578v1
	ASM2792550v1	ASM369716v2	ASM2792580v1
	ASM2792556v1	ASM396646v1	ASM2792582v1
	ASM2792568v1	ASM435836v1	ASM2792584v1
Salmonella enterica	ASM74305v1	ASM754v1	ASM1556573v1
	ASM155835v2	ASM2333072v1	ASM1602849v1
	ASM183155v2	ASM2333080v1	ASM1846004v1
	ASM332503v1	ASM2333084v1	ASM1933942v1
	ASM332513v1	ASM2436486v1	ASM2073634v1
	ASM332521v1	ASM2436490v1	ASM2147424v1
	ASM636533v1	ASM2436492v1	ASM2147428v1
	ASM696924v1	ASM2436494v1	ASM2253354v1
	ASM972991v1	ASM2436500v1	ASM2286996v1
	ASM1433415v1	ASM2953731v1	ASM2287068v1
Klebsiella pneumoniae	ASM1104559v1	ASM215672v1	ASM1982393v1
	ASM1106650v1	ASM215674v1	ASM2091180v1
	ASM1780983v1	ASM220219v1	ASM2269930v1
	ASM1812846v1	ASM276131v1	ASM2269934v1
	ASM1860434v1	ASM290300v1	ASM2274921v1
	ASM1982376v1	ASM636429v1	ASM2286966v1
	ASM1982380v1	ASM1018368v1	ASM2434689v1
	ASM1982383v1	ASM2794221v1	ASM2449616v1
	ASM1982387v1	ASM2847192v1	ASM2759564v1
	ASM1982391v1	ASM2920320v1	ASM2794219v1
